# Targeting the splicing factor SNRPB inhibits endometrial cancer progression by retaining the POLD1 intron

**DOI:** 10.1038/s12276-025-01407-2

**Published:** 2025-02-05

**Authors:** Yingwei Li, Zhongshao Chen, Huimin Xiao, Yanling Liu, Chen Zhao, Ning Yang, Cunzhong Yuan, Shi Yan, Peng Li

**Affiliations:** 1 Department of Obstetrics and Gynecology, Qilu Hospital of Shandong University. Medical Integration and Practice Center, Cheeloo College of Medicine, Shandong University, Ji’nan, China; 2 Department of Obstetrics and Gynecology, Qilu Hospital of Shandong University, Ji’nan, China

**Keywords:** Endometrial cancer, Endometrial cancer

## Abstract

Dysregulated alternative splicing has been closely linked to the initiation and progression of tumors. Nevertheless, the precise molecular mechanisms through which splicing factors regulate endometrial cancer progression are still not fully understood. This study demonstrated elevated expression of the splicing factor SNRPB in endometrial cancer samples. Furthermore, our findings indicate that high SNRPB expression is correlated with poor prognosis in patients with endometrial cancer. Functionally, SNRPB inhibition hindered the proliferative and metastatic capacities of endometrial cancer cells. Mechanistically, we revealed that SNRPB knockdown decreased POLD1 expression and that POLD1 intron 22 was retained after SNRPB silencing in endometrial cancer cells, as determined via RNA sequencing data analysis. The retained intron 22 of POLD1 created a premature termination codon, leading to the absence of amino acids 941–1,107 and the loss of the site of interaction with PCNA, which is essential for POLD1 enzyme activity. In addition, POLD1 depletion decreased the increase in the malignancy of endometrial cancer cells overexpressing SNRPB. Furthermore, miR-654-5p was found to bind directly to the 3′ untranslated region of SNRPB, resulting in SNRPB expression inhibition in endometrial cancer. Antisense oligonucleotide-mediated SNRPB inhibition led to a decrease in the growth capacity of a cell-derived xenograft model and a patient with endometrial cancer-derived xenograft model. Overall, SNRPB promotes the efficient splicing of POLD1 by regulating intron retention, ultimately contributing to high POLD1 expression in endometrial cancer. The oncogenic SNRPB–POLD1 axis is an interesting therapeutic target for endometrial cancer, and antisense oligonucleotide-mediated silencing of SNRPB may constitute a promising therapeutic approach for treating patients with endometrial cancer.

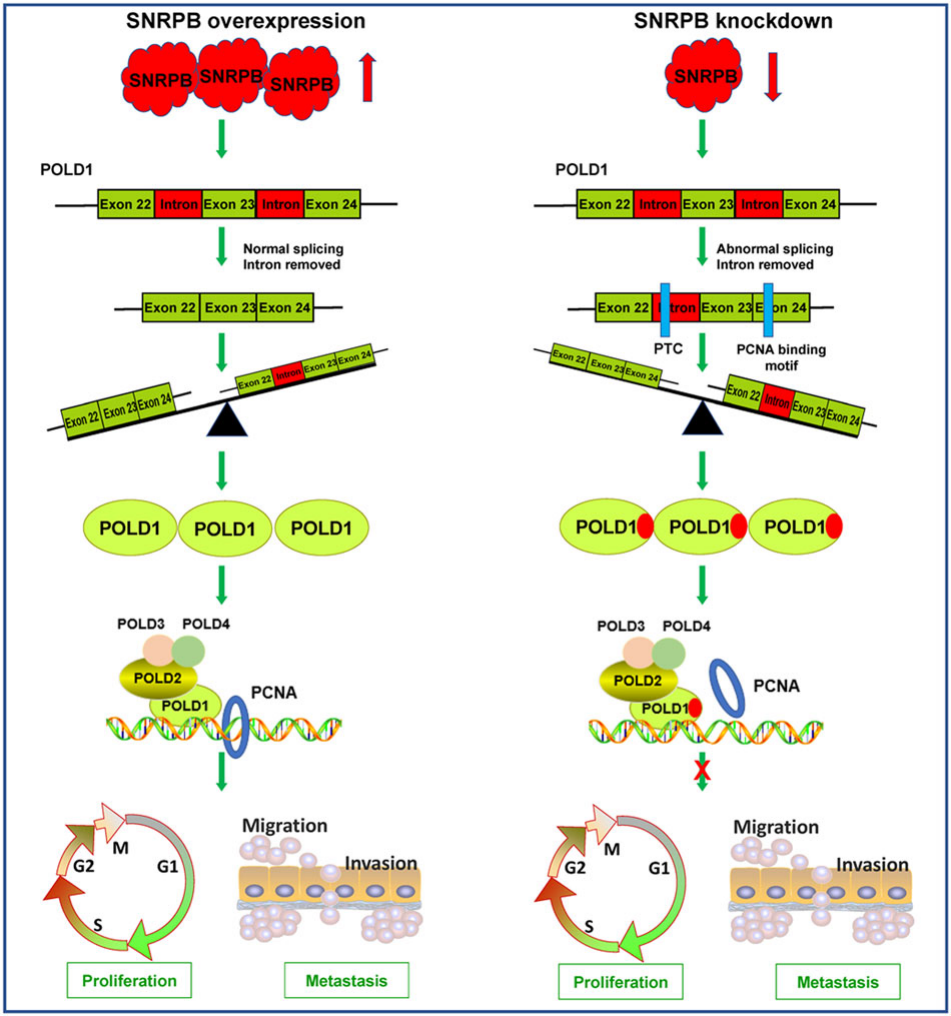

## Targeting SNRPB offers new hope for endometrial cancer

Endometrial cancer is a common cancer in women, with rising cases linked to obesity. This study focuses on a protein called SNRPB, which is involved in RNA splicing. SNRPB is found in high levels in endometrial cancer and is linked to poor outcomes. Researchers used various methods, including bioinformatics and lab experiments, to study SNRPB’s role. They found that reducing SNRPB levels slowed cancer cell growth and spread. They also discovered that SNRPB affects another protein, POLD1, which is important for DNA replication and repair. Lowering SNRPB led to changes in POLD1 that hindered cancer progression. This study suggests that targeting SNRPB with antisense oligonucleotides, which are short DNA or RNA molecules designed to block specific genes, could be a promising treatment strategy. This approach could lead to new therapies for endometrial cancer in the future.

This summary was initially drafted using artificial intelligence, then revised and fact-checked by the author.

## Introduction

In the USA, 66,200 women will be newly diagnosed with endometrial cancer in 2023, and 13,030 will die from it, making endometrial cancer one of the most common types of tumor in women^1^. A total of 69,000 new patients and 16,000 deaths were reported in China in 2015, resulting in a crude incidence rate of 10.28 cases per 100,000 individuals^2^. The increasing occurrence of obesity among women globally has led to an increase in the prevalence of endometrial cancer^3^. Patients with recurrent or metastatic endometrial cancer have few treatment options, and the survival rate is low, despite the good clinical prognosis for those with early-stage disease^4^. At present, the primary approach to treatment involves surgical procedures, radiation therapy, chemotherapy and progestogen therapy. However, women who desire to preserve their fertility cannot undergo a hysterectomy, and hormone therapy is often used to treat endometrial cancer in advanced stages (III or IV) or recurrent endometrial cancer after treatment. To develop novel therapeutic strategies for endometrial cancer, a deeper comprehension of the molecular characteristics that drive the development and progression of endometrial cancer is imperative^5^. Hence, it is of paramount importance to identify novel molecular biomarkers and potential therapeutic targets^6^.

Alternative splicing can regulate gene expression, which enables an individual gene to generate multiple different isoforms, increasing messenger RNA complexity and protein diversity^7^. Abnormal alternative splicing promotes the progression of malignant tumors^8^. According to recent research, the alternative processing of mRNAs may provide antigens specific to tumors that can be used for cancer vaccines and T cell therapies^9^. Alternative splicing is correlated with tumorigenesis and development of endometrial cancer cells. The splicing factor SF3B1 promotes proliferation and invasion by regulating KSR2 RNA maturation in endometrial cancer cells^5^. CircRAPGEF5 interacts with RBFOX2 to provide resistance to ferroptosis by inducing TFRC exon exclusion in endometrial cancer cells^10^. SNORA73B promotes endometrial cancer proliferation and metastasis by regulating MIB1 stability and RCC1 alternative splicing^11^. In endometrial cancer, alternative ANKHD1 transcripts facilitate the proliferation of tumor cells and inhibit their migration^12^. These findings suggest that alternative splicing may provide attractive therapeutic targets for cancer.

SNRPB is an essential component of the spliceosome for pre-mRNA splicing. Recent findings indicate that SNRPB abundance is elevated and correlated with a poor prognosis in various malignant tumors, which was determined via a pancancer analysis^13^. SNRPB promotes ovarian cancer growth and metastasis by inhibiting POLA1 and BRCA2 exon 3 skipping^14^. SNRPB facilitates cervical cancer and thyroid carcinoma progression by suppressing p53 expression^15,16^. SNRPB increases the ability of non‑small‑cell lung cancer cells to proliferate and metastasize by regulating RAB26 (ref. ^17^), and SNRPB also mediates the cellular response to cisplatin in non‑small‑cell lung cancer cells^18^. Based on functional genomics analysis, SNRPB performs oncogenic biological functions in glioblastoma^19^. SNRPB-mediated RNA splicing facilitates the growth and stemness of hepatocellular carcinoma cells^20^. Nevertheless, the underlying mechanism of elevated SNRPB expression levels in endometrial cancer remains unclear.

In this Article, we reveal that SNRPB was highly expressed in frozen or paraffin-embedded endometrial cancer tissues. The Kaplan–Meier plot survival analysis tool revealed that an elevated level of SNRPB was correlated with an unfavorable clinical outcome in patients with endometrial cancer. Functionally, SNRPB promoted the malignant characteristics of endometrial cancer cells. Antisense oligonucleotide (ASO)-mediated SNRPB silencing decreased the growth capacity of the cell-derived xenograft (CDX) model and patient with endometrial cancer-derived xenograft (PDX) model of endometrial cancer. Mechanistically, we found that SNRPB promotes efficient POLD1 splicing by regulating intron retention. Low expression of miR-654-5p contributed to the high expression of SNRPB in endometrial cancer cells through direct binding to the 3′-untranslated region (3′-UTR) of SNRPB. As a result, SNRPB might be useful in combating endometrial cancer as a novel therapeutic target. ASO-mediated silencing of SNRPB may provide an effective treatment strategy for treating patients with endometrial cancer.

## Materials and methods

### Bioinformatics analysis

GEPIA2 was used to identify the differentially expressed genes (DEGs) in endometrial cancer and endometrial samples^21^. TBtools was utilized to visualize the data presented in the heat map^20^. The effect of SNRPB on the survival rate of patients with endometrial cancer was assessed via an online survival analysis tool called the Kaplan–Meier plotter^22^. DAVID was used to perform Gene Ontology analysis^23^. rMATS was used to analyze differential alternative splicing events with mapped BAM files^24^. The RNA sequencing (RNA-seq) reads mapped to POLD1 were visualized via a Sashimi plot generated via Integrative Genomics Viewer (IGV).

### Tissue samples

Endometrial cancer and endometrial tissues were acquired from Qilu Hospital of Shandong University. Approval for the collection and utilization of clinical samples in this study was granted by the Ethical Committee of Qilu Hospital. The normal endothelial tissue utilized in this study was sourced from patients who underwent hysterectomy due to benign uterine conditions. The pathologic type of tumor tissue used in this study was endometrioid endometrial adenocarcinoma (Supplementary Table 3).

### Immunohistochemical staining

The sections from paraffin-embedded endometrial cancer tissues were deparaffinized and rehydrated, and then, microwave-mediated antigen retrieval treatment was performed in EDTA antigen retrieval buffer. To block unspecific antigens, goat serum was added after hydrogen peroxide was used to block endogenous peroxidase activity (Zhongshan Biotechnology Company). An overnight incubation with SNRPB antibody or Ki-67 was conducted on the sections at 4 °C. Under the microscope, the sections were stained with diaminobenzidine (Zhongshan Biotechnology Company). The intensity and extent of staining were used to determine the final score for each sample.

### Cell culture

Ishikawa cells were acquired from the European Collection of Authenticated Cell Cultures. AN3CA, HEC-1A and immortalized human endometrial stromal cells were obtained from the American Type Culture Collection (ATCC). Ishikawa cells were grown in Dulbecco’s modified Eagle medium (Gibco) supplemented with 10% fetal bovine serum (FBS) (Gibco). The AN3CA cells were cultured in modified Eagle medium (Gibco) supplemented with 10% FBS. McCoy’s 5A (Gibco), which was supplemented with 10% FBS, was used as the culture medium for the HEC-1A cells. The cell lines were cultivated in incubators containing 5% CO_2_ at 37 °C. Mycoplasma and short tandem repeats were assessed in all the cell lines.

### siRNA transfection and lentivirus infection

GenePharma designed short interfering RNA (siRNA) and ASO sequences that target SNRPB. The siRNA sequences that targeted POLD1 were acquired from a study conducted by Yejinpeng Wang^25^. The SNRPB overexpression (PCMV-SNRPB) and shRNA lentiviral vectors (PLKO.1-shSNRPB) were constructed by BioSune Biotechnology Company. The endometrial cancer cells were infected with lentiviral particles, and then, stably transfected cells were selected with puromycin. Lipofectamine 2000 (Invitrogen) was used for transfection according to the manufacturer’s instructions. The sequences of the siRNAs and ASOs used in this study are listed in Supplementary Tables 1 and 2.

### RNA isolation and qPCR assays

We extracted total RNA with TRIzol reagent (Invitrogen, USA), and total RNA was reverse-transcribed into cDNA via an Evo M-MLV RT kit (Accurate Biology, China). QuantStudio 3 (Applied Biosystems) was utilized to examine target gene expression via SYBR Green (Accurate Biology, China). GAPDH was used as a control. The quantitative polymerase chain reaction (qPCR) results were analyzed via the 2^−ΔΔCT^ method.

### Western blotting assay

The protein extracts were acquired by lysing samples in RIPA buffer (Beyotime Biotechnology). A BCA protein assay kit (Merck Millipore) was used to determine protein concentrations. After the protein lysates were separated via 10% SDS–polyacrylamide gel electrophoresis, the proteins were subsequently transferred onto polyvinylidene difluoride membranes (Merck Millipore). After nonspecific antigens were blocked with 5% skim milk, the samples were incubated with primary antibodies at 4 °C overnight. The secondary antibodies labeled with HRP and either anti-rabbit or anti-mouse IgGs were used. Following washing TBS with Tween-20 (TBST), the protein bands were analyzed via enhanced chemiluminescence (Merck Millipore). β-actin served as an endogenous control.

### Antibodies

For western blotting, the following antibodies were utilized: SNRPB (MA5-13449, Invitrogen), POLD1 (ab186407, Abcam), Flag antibody (F1804, Sigma-Aldrich) and β-actin (A5441, Sigma-Aldrich). Immunohistochemistry (IHC) staining was performed using SNRPB (MA5-13449, Invitrogen) and Ki-67 (no. 9027, Cell Signaling Technology) antibodies. An RNA immunoprecipitation assay was performed using an anti-Flag antibody (F1804, Sigma-Aldrich).

### Cell proliferation and clonogenic assays

The endometrial cell growth was evaluated via the 3-(4,5-dimethylthiazol-2-yl)-2,5-diphenyltetrazolium bromide (MTT) (Beyotime Biotechnology) assay. In 96-well plates, 800–1200 cells were seeded per well, and 20 μl of MTT was added. Dimethylsulfoxide was used to dissolve the purple formazan crystals (Beyotime Biotechnology) after the culture medium was removed. The absorbance was measured via a Varioskan Flash reader (Bio-Rad).

A clonogenic assay was utilized to assess the capacity of an individual cell to proliferate and establish a colony. To assess the colony formation capacity, 1000–1200 cells were placed in six-well dishes and incubated for a period of 10–14 days. After methanol fixation and the application of crystal violet stain, the viability of the colonies was determined by counting those containing more than 50 cells.

### EdU assay

The proliferative status of the cells was evaluated via an ethynyldeoxyuridine (EdU) assay kit (Beyotime), which measures the proportion of cells undergoing DNA replication. The EdU incorporation rate was determined by calculating the ratio of EdU-positive cells to those stained with Hoechst 33342.

### Cell cycle and apoptosis assays

For the cell cycle assay, the cells were stained with propidium iodide following the protocol (CCS012, MultiSciences Biotech). The distribution of cell cycle phases across different groups was subsequently assessed via the Beckman CytoFLEX FCM (Beckman Coulter), with data analysis performed via ModFit LT software. For the apoptosis assays, the cells were resuspended in 1× binding buffer (556547, BD Bioscience, Franklin Lakes) and stained with FITC Annexin V and propidium iodide. The stained cells were then analyzed via CytoFLEX software.

### Migration and invasion assays

Transwell assays were used to assess the migration and invasion capacity of endometrial cancer cells. The upper compartment of the chamber contained cell suspensions without serum. In the lower chamber, culture medium containing 20% FBS was added. The plates were incubated in a 37 °C incubator. Following immersion of the chamber in methanol, the lower surface of the cells was dyed with crystal violet after they had migrated. The invasive capacity of the cells was assessed via a transwell assay with Matrigel. The cells that migrated or invaded were counted with a microscope.

### Nude mouse subcutaneous xenograft assay

We purchased female nude mice (BALB/c, 4 weeks old) from GemPharmatech. The nude mice were subcutaneously injected with Ishikawa cells transfected with PLKO.1-shSNRPB or PLKO.1-ctrl (*n* = 5 for each group). The mice were raised in an Specific Pathogen Free (SPF) environment for 2 weeks. Following euthanasia and dissection of the mice, the tumors were photographed, and the weights were measured. By measuring the length and width of the tumor, the tumor volume was determined via the formula: length × width^2^/2). The Ethics Committee of Qilu Hospital of Shandong University granted approval for the animal experiments.

### PDX model

Fresh endometrial cancer tissues were obtained from Qilu Hospital of Shandong University and subsequently fragmented for subcutaneous implantation into NCG mice (GemPharmatech) to establish a PDX model. PDX tumors were successfully passaged by serial transplantation in NCG mice for further studies. The mice were randomly divided into the SNRPB-ASO group (*n* = 5) and the ASO-NC group (*n* = 5) and received intratumoral injections of SNRPB-ASO or ASO-NC every other day. The mice were subsequently euthanized, and the weights and volumes of the tumors were assessed. The sequences of the ASOs used in this study are listed in Supplementary Table 2.

### RNA-seq analysis

Transient transfection of Ishikawa cells was performed with si-SNRPB and si-NC, followed by isolation of total RNA via TRIzol reagent after 48 h. RNA-seq was conducted at BioSune Biotechnology Company, which is in China. The raw data were uploaded to the GEO database (GSE279656). DEGs were identified based on the criteria of |log_2_ fold change| ≥0.58 and *q* < 0.05.

### RNA immunoprecipitation assay

We carried out RNA immunoprecipitation assays in Ishikawa cells overexpressing Flag-SNRPB via an anti-Flag antibody. We followed the procedure in accordance with the instructions of the Millipore (17–700) kit. The reverse transcription of immunoprecipitated RNA was utilized for subsequent qPCR and RT‒PCR analysis.

### Statistical analysis

The experiments were carried out with at least three replicates. The data are presented as the mean ± standard error of the mean. Unpaired two-tailed Student’s *t*-tests were used to assess the statistical significance of the differences. GraphPad Prism 5 was used to conduct the statistical analysis. The differences were considered statistically significant when *P* < 0.05.

## Results

### SNRPB is a critical driver of endometrial cancer

**Fig. 1:**
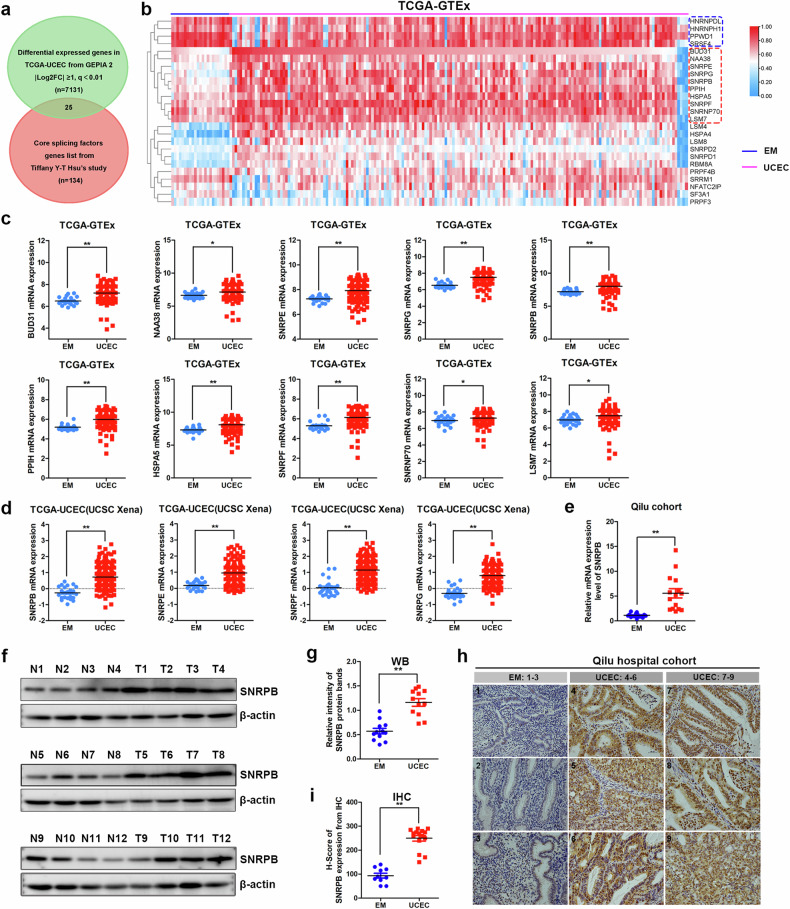
SNRPB is a critical driver of endometrial cancer. **a** A comparative analysis of DEGs between endometrial cancer and normal endometria from GEPIA2 and 134 core splicing factors from Tiffany’s study revealed 25 critical core splicing factors, including 15 splicing factors with upregulated expression and 10 splicing factors with downregulated expression in endometrial cancer tissues. **b** A heat map analysis of 25 differentially expressed core splicing factors in endometrial cancer tissues (*n* = 181) compared with normal endometrial tissues (*n* = 23) in TCGA-GTEx from UCSC Xena. **c** A differential expression analysis of BUD31, NAA38, SNRPE, SNRPG, SNRPB, PPIH, HSPA5, SNRPF, SNRNP70 and LSM7 between endometrial cancer (*n* = 181) and normal endometrial tissues (*n* = 23) via data from TCGA-GTEx. **d** A differential expression analysis of SNRPB, SNRPE, SNRPF and SNRPG between endometrial cancer (*n* = 177) and normal endometrial tissues (*n* = 24) using data from TCGA-UCEC. **e** A qPCR analysis of SNRPB mRNA expression in fresh-frozen tissues from patients with endometrial cancer (*n* = 15) and normal endometria (*n* = 15). **f** A western blotting analysis of SNRPB protein expression in fresh-frozen tissues from patients with endometrial cancer (T, *n* = 12) and normal endometria (N, *n* = 12). **g** A quantitative analysis of the SNRPB protein bands derived from **f**. **h** A representative images of immunohistochemical staining for SNRPB in endometrial cancer tissues and normal endometrial tissues. **i**, Immunohistochemical histological scores (*H*-scores) of the SNRPB protein in endometrial cancer tissues (*n* = 15) and normal endometrial tissues (*n* = 10). The *P* values were obtained via unpaired *t*-tests. *P < 0.05, **P < 0.01.

The DEGs between endometrial cancer and endometrial tissues from the uterine corpus endometrial carcinoma (TCGA-UCEC) cohort were obtained from GEPIA2. The overlapping analysis of DEGs in TCGA-UCEC and core splicing factors^26^ revealed 15 splicing factors that were upregulated in endometrial cancer tissues, whereas 10 splicing factors were downregulated. A heat map analysis of the 25 splicing factors that are differentially expressed between endometrial cancer and normal endometrial tissues in the TCGA-GTEx datasets from UCSC Xena was subsequently performed. The findings revealed that four splicing factors, HNRNPDL, HNRNPH1, PPWD1 and SRSF4, were significantly upregulated in normal endometrial tissues, whereas 10 splicing factors, BUD31, NAA38, SNRPE, SNRPG, SNRPB, PPIH, HSPA5, SNRPF, SNRNP70 and LSM7, were highly expressed in endometrial cancer tissues. Among the highly expressed splicing factors in cancer tissues, four were identified as Sm proteins (Fig. 1a, b). Dysregulation and mutation of Sm proteins have been associated with various human diseases, including cancer^27^. The differential expression of HNRNPDL, HNRNPH1, PPWD1 and SRSF4 in endometrial cancer compared with normal endometrial (EM) tissues is depicted in Supplementary Fig. 1a. The relative expression levels of BUD31, NAA38, SNRPE, SNRPG, SNRPB, PPIH, HSPA5, SNRPF, SNRNP70, and LSM7 are shown in Fig. 1c. These results suggest a potential role for Sm proteins in the pathogenesis and progression of endometrial cancer. Differential expression analysis of SNRPB, SNRPE, SNRPF and SNRPG proved that these splicing factors were overexpressed in endometrial cancer tissues via data from TCGA-UCEC (Fig. 1d). We demonstrated that the splicing factor SNRPB promoted the malignant progression of ovarian cancer cells through regulating POLA1 and BRCA2 exon skipping in our previous study^14^. In this study, we focus on the biological function and related regulatory mechanisms of SNRPB in endometrial cancer.

To confirm SNRPB mRNA and protein expression levels in endometrial cancer, we utilized qPCR and western blotting to assess SNRPB expression in fresh-frozen tissues from patients with endometrial cancer and normal endometria. The results revealed significant overexpression of SNRPB in the endometrial cancer samples (Fig. 1e–g). Similarly, immunohistochemical staining revealed that the intensity of SNRPB staining was significantly greater in endometrial cancer tissue than in normal endometrial tissue (Fig. 1h, i). Survival analysis via Kaplan‒Meier plotter revealed that elevated SNRPB levels were linked to unfavorable overall survival (OS) and relapse-free survival among individuals with endometrial cancer (Supplementary Fig. 1b, c). These data imply that SNRPB is overexpressed in endometrial cancer and that SNRPB could play a crucial role in the progression of endometrial cancer.

### SNRPB promotes the proliferative and metastatic capacities of endometrial cancer cells in vitro

**Fig. 2:**
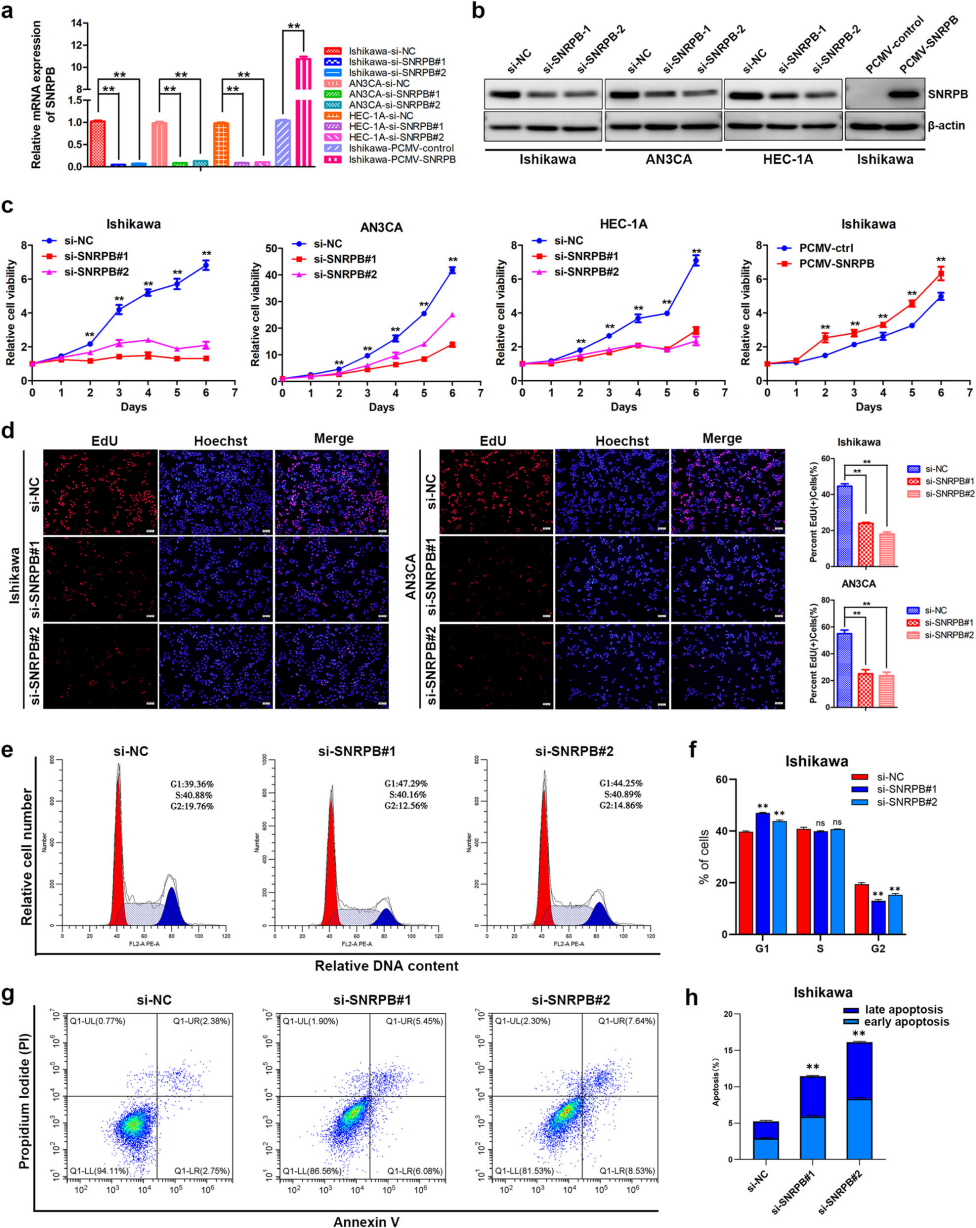
SNRPB promotes the proliferation and metastasis of endometrial cancer cells in vitro. **a** The overexpression and knockdown efficiencies of SNRPB in endometrial cancer cells were verified via qPCR (*n* = 3 biologically independent samples). **b** The overexpression and knockdown efficiencies of SNRPB in endometrial cancer cells were verified via western blotting (*n* = 3 biologically independent samples). **c** A MTT assay was used to evaluate the impact of SNRPB knockdown in Ishikawa, AN3CA and HEC-1A cells and SNRPB overexpression in Ishikawa cells on the proliferation of endometrial cancer cells (*n* = 3 biologically independent samples). **d** The fraction of DNA-replicating cells was reduced by SNRPB silencing in Ishikawa and AN3CA cells, which were subjected to an EdU incorporation assay (*n* = 3 biologically independent samples). **e** The flow cytometric analysis of the effect of SNRPB silencing on the cell cycle distribution of Ishikawa cells. **f** Quantification of the percentage of cells in different phases from (**e**). **g** A flow cytometric analysis of the impact of SNRPB inhibition on the apoptosis of Ishikawa cells. **h** Quantification of the percentage of apoptosis cells from (**g**). The *P* value was obtained via an unpaired *t* test. *P < 0.05, **P < 0.01.

To investigate the biological function of SNRPB in endometrial cancer, siRNAs against SNRPB were used to silence SNRPB expression in Ishikawa, AN3CA and HEC-1A cells via transient transfection. The cells with stable SNRPB overexpression were generated via lentiviral vector transfection of Ishikawa cells. The overexpression and interference efficiencies of SNRPB in endometrial cancer cells were verified via qPCR and western blotting (Fig. 2a, b and Supplementary Fig. 1d).

MTT and colony formation assays were performed to evaluate the impact of SNRPB on the growth speed and viability of endometrial cancer cells. Silencing SNRPB significantly suppressed the proliferation of Ishikawa, AN3CA and HEC-1A cells, as demonstrated by the MTT assay (Fig. 2c). Conversely, SNRPB overexpression stimulated the growth of Ishikawa cells (Fig. 2c). The colony formation assay also demonstrated that SNRPB silencing reduced the colony formation ability of Ishikawa, AN3CA and HEC-1A cells, whereas ectopic SNRPB expression increased the clonogenicity of Ishikawa cells (Supplementary Fig. 1e). EdU incorporation assays revealed that the fraction of DNA-replicating cells was reduced by silencing SNRPB expression in Ishikawa, AN3CA and HEC-1A cells (Fig. 2d and Supplementary Fig. 1f). Flow cytometry analysis demonstrated that SNRPB knockdown resulted in cell cycle arrest at the G1 phase and induced the apoptosis of Ishikawa cells (Fig. 2e–h). The metastatic capacity of Ishikawa, AN3CA and HEC-1A cells was assessed via Transwell migration assays and Matrigel-coated Transwell invasion assays. The findings demonstrated that the reduction in SNRPB resulted in a decrease in the metastatic capacity of endometrial cancer cells, and SNRPB overexpression promoted the metastatic ability of Ishikawa cells (Supplementary Fig. 2a, b). These results indicate that the splicing factor SNRPB contributes to tumorigenesis by promoting the growth speed and metastasis ability of endometrial cancer cells in vitro.

### SNRPB silencing inhibits the subcutaneous tumorigenic ability of endometrial cancer cells in nude mice

**Fig. 3:**
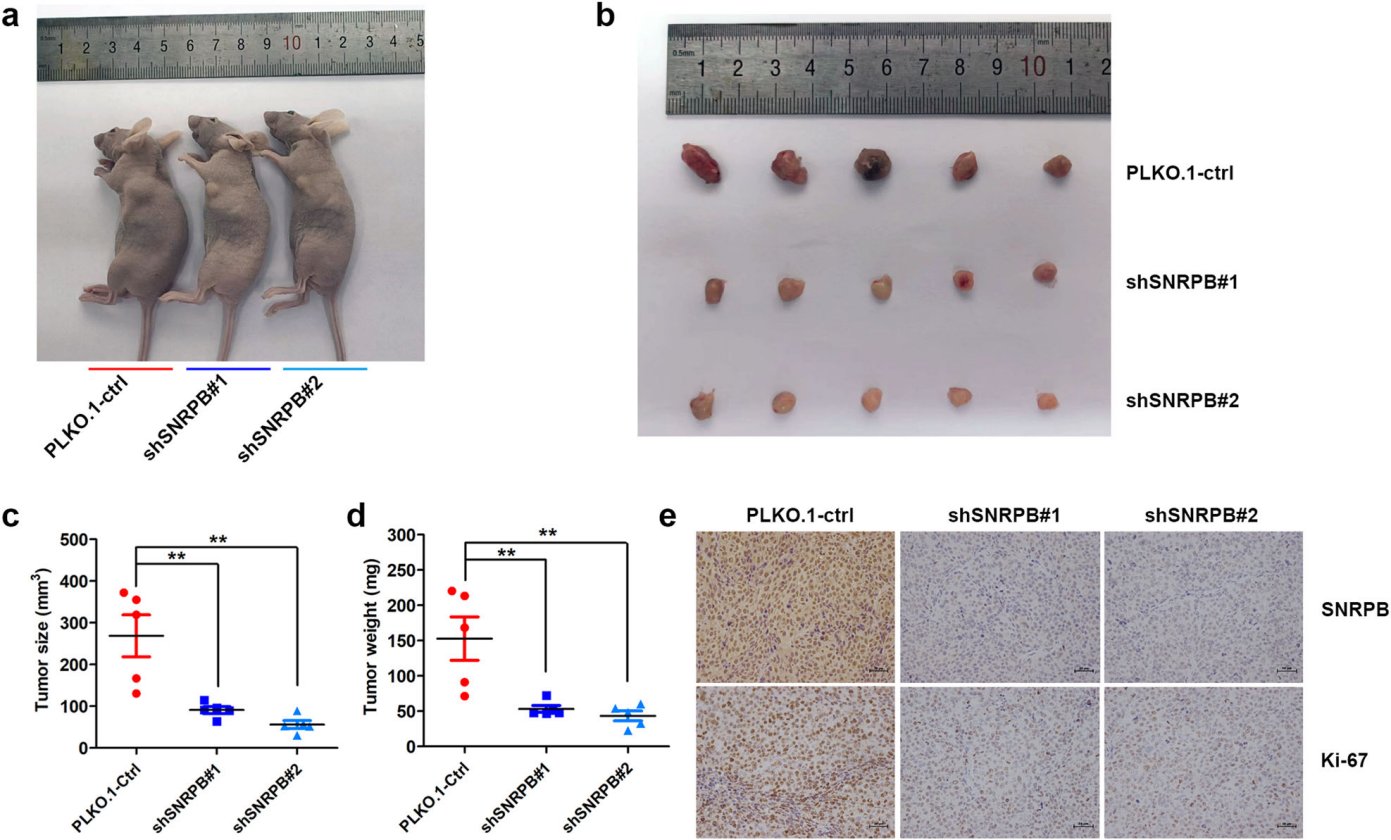
SNRPB silencing inhibits the tumorigenic ability of endometrial cancer cells in vivo. **a** Representative images of endometrial tumors in nude mice in the shRNA against SNRPB group (PLKO.1-shSNRPB#1 and PLKO.1-shSNRPB#2) and PLKO.1-control group. **b** Photographs of subcutaneous tumors removed from the sacrificed model mice in the PLKO.1-shSNRPB group and PLKO.1-control group (*n* = 5 mice per group). **c** The volume of the subcutaneous tumors in the PLKO.1-shSNRPB group and PLKO.1-control group were measured and calculated. **d** The weight of the subcutaneous tumors in the PLKO.1-shSNRPB group and PLKO.1-control group were measured and calculated. **e** IHC staining of SNRPB and Ki-67 in the PLKO.1-shSNRPB group and PLKO.1-control group. The *P* value was obtained via an unpaired *t*-test. *P < 0.05, **P < 0.01.

To determine the impact of SNRPB on the growth speed of endometrial cancer cells in vivo, we conducted a subcutaneous tumor xenograft experiment. Ishikawa cells were transduced with lentiviral particles expressing PLKO.1-shRNA against SNRPB (PLKO.1-shSNRPB#1 and PLKO.1-shSNRPB#2) and PLKO.1-ctrl shRNA. These cells were subsequently injected subcutaneously into the axillary fossa of nude mice (*n* = 5 for each group) (Fig. 3a). The nude mice were euthanized, and the subcutaneous tumors were gently removed, photographed and weighed 2 weeks after implantation. The findings indicated that the volume and weight of tumors in the PLKO.1-shSNRPB group were notably lower than those in the PLKO.1-ctrl group (Fig. 3b–d). In addition, we detected Ki-67 expression and found that Ki-67 expression was significantly decreased after SNRPB inhibition (Fig. 3e). These results suggested that the growth of tumors was significantly suppressed after SNRPB silencing in endometrial cancer cells. Collectively, these results indicate that SNRPB depletion hinders tumor growth by reducing the proliferative ability of endometrial cancer cells in vivo.

### Low expression of miR-654-5p contributes to high expression of SNRPB in endometrial cancer

**Fig. 4:**
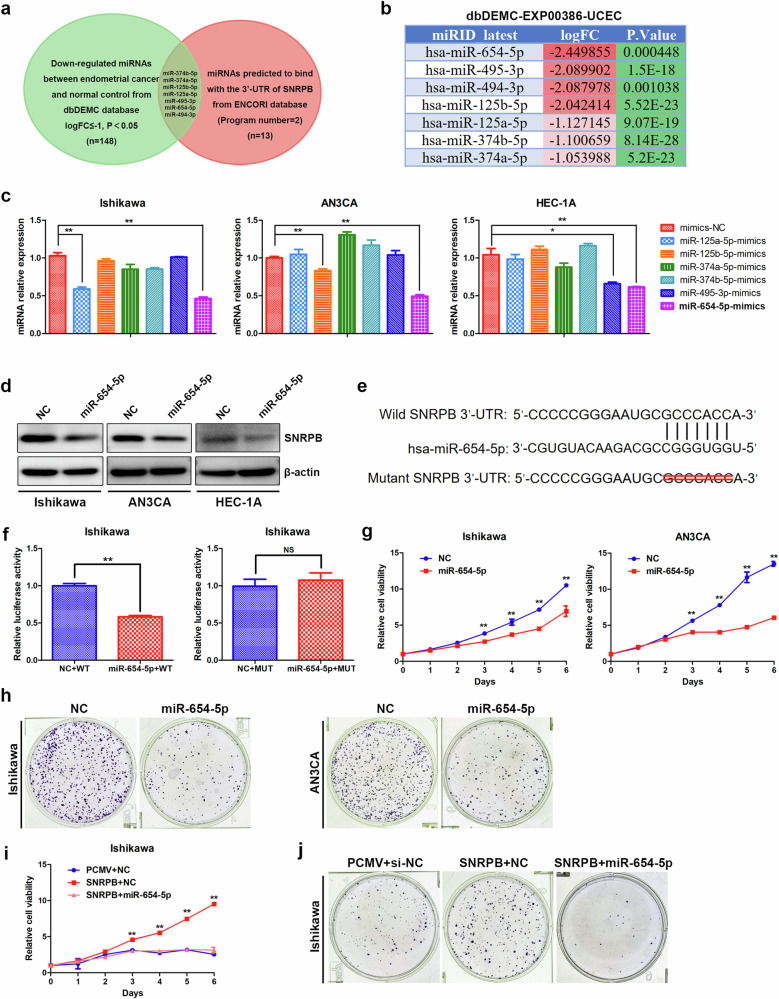
Low expression of miR-654-5p contributes to the high expression of SNRPB in endometrial cancer. **a** A Venn diagram demonstrating the intersection of downregulated miRNAs (TCGA miRNA-seq from the dbDEMC database) and miRNAs predicted to bind to the SNRPB 3′-UTR in the ENCORI database. **b** Differential expression levels of the seven miRNAs between endometrial cancer and normal endothelium. **c** The effects of miRNAs (miR-374b-5p, miR-374a-5p, miR-125b-5p, miR-125a-5p, miR-495-3p and miR-654-5p) on SNRPB mRNA expression were analyzed via a qPCR assay (*n* = 3 biologically independent samples). **d** The effects of the miR-654-5p mimics on SNRPB protein expression were analyzed via western blotting. **e** Wild-type and mutant luciferase reporter plasmids containing the SNRPB 3′-UTR. **f** A dual-luciferase reporter gene assay was used to determine the effect of miR-654-5p mimics on the luciferase activity of the SNRPB 3′-UTR group in Ishikawa cells (*n* = 3 biologically independent samples). **g** The effects of the miR-654-5p mimics on the proliferation capacity of Ishikawa and AN3CA cells were determinedvia MTT assay (*n* = 3 biologically independent samples). **h** The effects of the miR-654-5p mimics on the proliferation capacity of Ishikawa and AN3CA cells were determined via colony formation assay (*n* = 3 biologically independent samples). **i** MTT assay were used to evaluate the effects of the miR-654-5p mimics on the proliferative ability of Ishikawa cells overexpressing SNRPB (*n* = 3 biologically independent samples). **j** The colony formation assays were used to evaluate the effects of the miR-654-5p mimics on the proliferative ability of Ishikawa cells overexpressing SNRPB (*n* = 3 biologically independent samples). The *P* values were obtained via an unpaired *t*-test. *P < 0.05, **P < 0.01.

Post-transcriptional regulation may be affected by miRNAs, which bind to the 3′-UTR of target genes to reduce their expression. To identify the key miRNAs regulating SNRPB expression, we first used the ENCORI database to predict miRNAs that could bind to the SNRPB 3′-UTR, resulting in the identification of 13 miRNAs (program number 2). We subsequently analyzed differentially expressed miRNAs between endometrial cancer and normal endometrial tissues via the dbDEMC database (TCGA miRNA-seq). A total of 148 genes were found to be downregulated in endometrial cancer tissues compared with normal endometrial tissues (logFC ≤−1, *P* < 0.05). Through the analysis of the intersection of downregulated miRNAs and miRNAs predicted to bind with the SNRPB 3′-UTR, seven miRNAs were identified, namely, miR-374b-5p, miR-374a-5p, miR-125b-5p, miR-125a-5p, miR-495-3p, miR-654-5p and miR-494-3p (Fig. 4a). The differential expression levels of these seven miRNAs between endometrial cancer and normal endothelium samples are depicted in Fig. 4b. miR-494-3p has been shown to be significantly upregulated in endometrial cancer tissues and to play a role in promoting disease progression^28^. In this study, we investigate the regulatory effects of six other miRNAs on the expression of SNRPB in endometrial cancer cells. Our findings indicate that only miR-654-5p was able to significantly downregulate SNRPB mRNA expression in three different endometrial cancer cell lines following the overexpression of the six miRNAs (Fig. 4c). The western blotting results further confirmed that the overexpression of miR-654-5p led to a significant decrease in SNRPB protein expression in endometrial cancer cells (Fig. 4d and Supplementary Fig. 2c). We constructed wild-type and mutant luciferase reporter plasmids containing the SNRPB 3′-UTR (Fig. 4e). The overexpression of miR-654-5p led to a decrease in luciferase activity in the wild-type SNRPB 3′-UTR group of Ishikawa cells (Fig. 4f). Conversely, upon mutation of the binding site, the luciferase activity remained unchanged following miR-654-5p overexpression in Ishikawa cells (Fig. 4f). These findings indicate that miR-654-5p mediates the reduction in SNRPB expression by directly binding to its 3′-UTR in endometrial cancer cells.

MTT proliferation and colony formation assays were used to evaluate the impact of miR-654-5p overexpression on the viability of endometrial cancer cells. The findings demonstrated that the overexpression of miR-654-5p resulted in a reduction in the growth rate and colony formation capacity of Ishikawa and AN3CA cells (Fig. 4g, h). Subsequent experiments confirmed that the overexpression of miR-654-5p counteracted the proliferative effect of SNRPB on endometrial cancer cells (Fig. 4i, j). In conclusion, these results reveal that downregulation of miR-654-5p contributes to the high expression of SNRPB in endometrial cancer cells through direct binding to its 3′-UTR.

### POLD1 is a critical downstream target of SNRPB in endometrial cancer cells

**Fig. 5:**
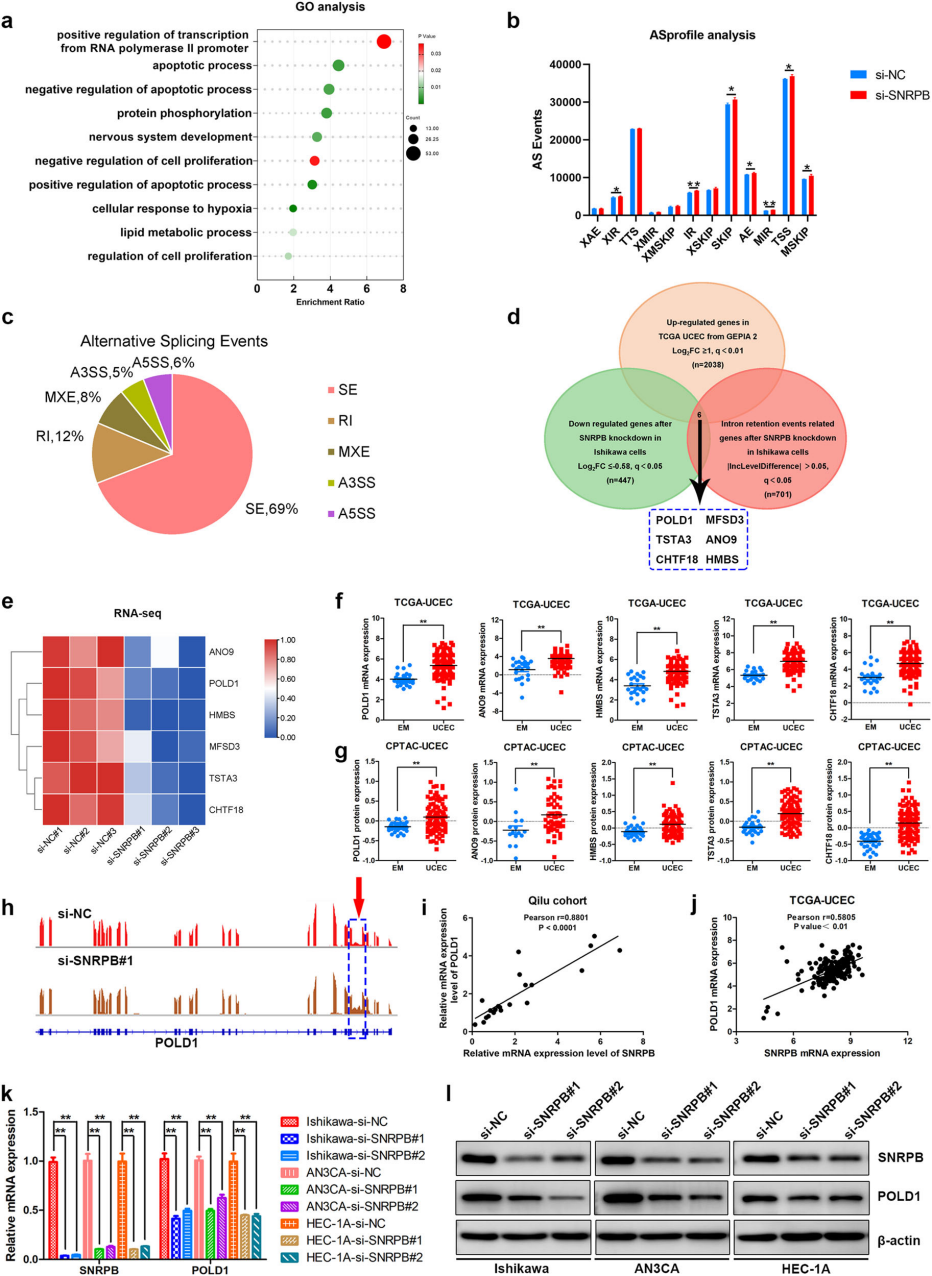
POLD1 is the critical downstream target of SNRPB in endometrial cancer cells. **a** GO ontology analysis of the DEGs identified by RNA-seq after SNRPB knockdown in Ishikawa cells. **b** ASprofile was used to compare the 12 basic AS events from the RNA-seq data after SNRPB knockdown in Ishikawa cells. **c** rMATS was used to define AS events and identify crucial alternative splicing events following SNRPB depletion in Ishikawa cells. **d** The downstream crucial target genes were selected by comparing the genes affected by differential intron retention AS events, downregulated DEGs after SNRPB knockdown and genes with upregulated expression in the TCGA-UCEC cohort. **e** A heat map of RNA-seq data demonstrating the selected crucial target genes. **f** The mRNA expression levels of selected target genes were analyzed in endometrial cancer and normal endometrial tissues from the TCGA-UCEC (endometrial cancer (*n* = 181); normal endometrium (*n* = 23)). **g** The protein expression levels of selected target genes were analyzed in endometrial cancer and normal endometrial tissues from the CPTAC (endometrial cancer (*n* = 100); normal endometrium (*n* = 31)) cohort. **h** RNA-seq reads mapped to POLD1 cells after SNRPB knockdown in Ishikawa cells were visualized via an IGV/Sashimi plot. **i** A qPCR analysis of the relationship between SNRPB and POLD1 mRNA expression in fresh-frozen endometrial cancer tissues (*n* = 22) from the Qilu cohort. **j** A correlation analysis of SNRPB and POLD1 expression in the TCGA-UCEC cohort (*n* = 181). **k** qPCR was used to assess POLD1 expression levels after SNRPB knockdown in endometrial cancer cells. l Western blotting was used to assess POLD1 expression levels after SNRPB knockdown in endometrial cancer cells. The *P* value was obtained via an unpaired *t*-test. *P < 0.05, **P < 0.01.

To study the molecular mechanisms by which SNRPB performs its oncogenic functions, SNRPB expression was silenced via specific siRNAs, and RNA-seq was subsequently performed in Ishikawa cells. RNA-seq analysis revealed 885 DEGs, including 438 upregulated and 447 downregulated genes, as defined by a |log_2_FC| ≥0.58 and adjusted *P* < 0.05. Gene Ontology (GO) ontology analysis of DEGs after SNRPB knockdown in Ishikawa cells identified via RNA-seq revealed that these DEGs were associated mainly with transcription regulation, apoptosis, protein phosphorylation, hypoxia and proliferation (Fig. 5a).

As SNRPB is a core splicing factor, ASprofile was used to compare 12 basic alternative splicing (AS) events after SNRPB silencing in Ishikawa cells via RNA-seq data. Among the AS events identified, the differences in the number of approximate IR (XIR), intron retention (IR), skipped exon (SKIP), alternative exon ends (AE), multi-IR (MIR), transcription start site (TSS) and multiexon SKIP (MSKIP) events were significant (Fig. 5b). Thus, there was an obvious correlation between intron retention and SNRPB expression levels. Next, to identify crucial alternative splicing events following SNRPB depletion, rMATS was used to define AS events with |IncLevelDifference| >0.05 and *q* < 0.05 (ref. ^10^), and 5,742 AS events were identified. Among them, exon skipping had the highest percentage (69.14%), followed by intron retention (12.21%) (Fig. 5c).

By comparing the genes affected by differential intron retention AS events, the downregulated DEGs after SNRPB knockdown and the genes whose expression was upregulated in the TCGA-UCEC cohort, we identified six crucial target genes (POLD1, MFSD3, TSTA3, ANO9, CHTF18 and HMBS), as shown in Fig. 5d. The mRNA expression of the selected six target genes was analyzed via the RNA-seq data of SNRPB-knockdown Ishikawa cells (Fig. 5e). The expression levels of the six target genes were analyzed in endometrial cancer and normal endometrial tissues in the TCGA-UCEC and CPTAC cohorts (MFSD3 expression data were not available in the datasets); the results revealed that these selected target genes were overexpressed in endometrial cancer tissues (Fig. 5f, g and Supplementary Fig. 2d). POLD1 plays a significant role in DNA replication and repair, and it also contributes significantly to the initiation and progression of tumors^29^. Differential expression of POLD1 was detected via qPCR and western blotting in freshly frozen endometrial cancer and normal endometrial tissues, which revealed that POLD1 was significantly highly expressed in endometrial cancer (Supplementary Fig. 2e–g). Therefore, POLD1 was selected as an important downstream target of SNRPB in endometrial cancer. The RNA-seq reads mapped to POLD1 after SNRPB depletion in Ishikawa cells were visualized via a Sashimi plot, which revealed intron 22 retention in POLD1 pre-mRNA (Fig. 5h). Furthermore, the SNRPB and POLD1 mRNA levels were simultaneously detected in fresh-frozen tissues via qPCR, and Pearson’s correlation analysis was performed. SNRPB was significantly positively correlated with POLD1 expression in endometrial cancer (Pearson *r* = 0.8801) (Fig. 5i). In the TCGA-UCEC cohort, the correlation analysis also revealed a positive association between SNRPB and POLD1 expression (Pearson *r* = 0.5805) (Fig. 5j). To validate SNRPB regulation of POLD1 expression, POLD1 expression levels were assessed after SNRPB knockdown via qPCR and western blotting. The results suggested that SNRPB inhibition decreased POLD1 expression in endometrial cancer cells (Fig. 5k, l and Supplementary Fig. 2h). These findings indicated that the splicing factor SNRPB regulated intron retention and the expression level of POLD1, and POLD1 was identified as the crucial downstream target of SNRPB in endometrial cancer cells.

### High expression of POLD1 is correlated with adverse clinical outcomes in patients with endometrial cancer

The POLD1 mutation rate was 8% in patients with endometrial cancer according to the TCGA PanCancer atlas (Supplementary Fig. 3a). We also evaluated the effect of POLD1 mutation on patient survival and found that POLD1 mutation was positively correlated with Progression Free Survival (PFS), Disease Specific Survival (DSS) and OS in patients with endometrial cancer (Supplementary Fig. 3b–d). We interrogated the TCGA PanCancer atlas database to determine POLD1 mRNA expression in patients with endometrial cancer. The ratio of patients with high POLD1 mRNA expression was 8%, and high POLD1 expression was associated with worse Progression Free Survival (PFS) in patients with endometrial cancer (Supplementary Fig. 3e, f). The correlation between POLD1 mRNA expression and clinical outcomes was examined via the Kaplan–Meier plot website, and high POLD1 expression was also correlated with poor OS and relapse-free survival in patients with endometrial cancer (Supplementary Fig. 3g, h). These findings suggest that POLD1 mutation is an important factor contributing to favorable prognosis and that high POLD1 expression is an adverse prognostic biomarker in patients with endometrial cancer.

### POLD1 knockdown suppressed the malignant behavior of endometrial cancer

**Fig. 6:**
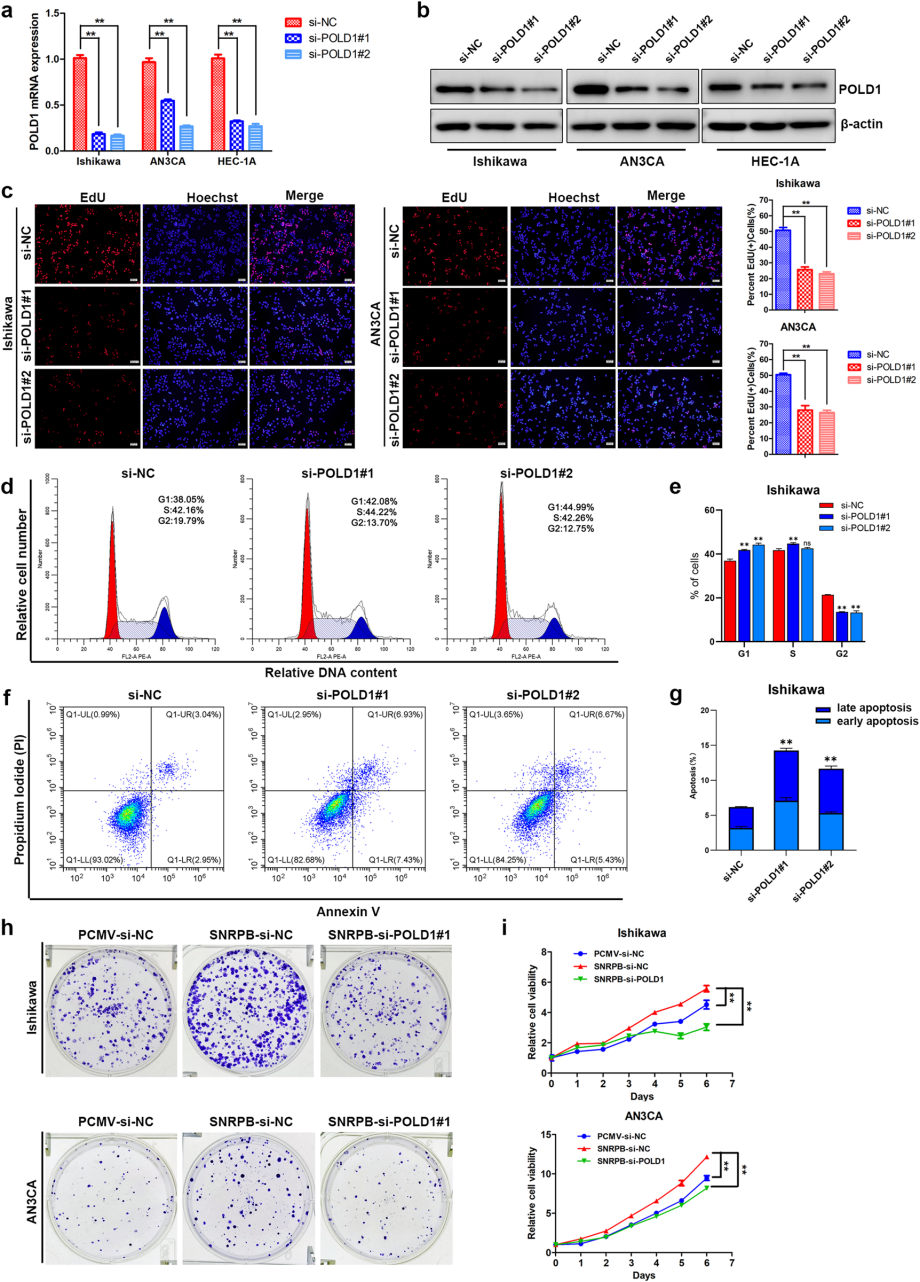
POLD1 knockdown suppressed the malignant behavior of endometrial cancer cells. **a** qPCR was conducted to determine the interference efficiency of POLD1 siRNA in Ishikawa, AN3CA and HEC-1A cells (*n* = 3 biologically independent samples). **b** Western blotting was conducted to determine the interference efficiency of POLD1 siRNA in Ishikawa, AN3CA and HEC-1A cells (*n* = 3 biologically independent samples). **c** The fraction of DNA-replicating cells was reduced by silencing POLD1 in Ishikawa and AN3CA cells, which were subjected to an EdU incorporation assay (*n* = 3 biologically independent samples). **d** A flow cytometric analysis of the effect of POLD1 silencing on the cell cycle distribution of Ishikawa cells. **e** Quantification of the percentage of cells in different phases from (**d**). **f**, **g** A flow cytometric analysis of the impact of POLD1 inhibition on the apoptosis of Ishikawa cells. **h** The colony formation assay was applied to investigate the effects of POLD1 knockdown on the clonogenic activity of SNRPB-overexpressing Ishikawa and AN3CA cells (*n* = 3 biologically independent samples). **i** MTT assay was applied to investigate the effects of POLD1 knockdown on the proliferation of SNRPB-overexpressing Ishikawa and AN3CA cells (*n* = 3 biologically independent samples). The *P* value was obtained via an unpaired *t*-test. *P < 0.05, **P < 0.01.

To investigate the role of POLD1 in endometrial cancer cells, we used siRNA to deplete POLD1 in Ishikawa, AN3CA and HEC-1A cells. We conducted qPCR and western blotting experiments to determine the interference efficiency of POLD1 siRNA (Fig. 6a, b and Supplementary Fig. 4a). A growth curve assay revealed that POLD1 knockdown decreased the growth speed of Ishikawa, AN3CA and HEC-1A cells (Supplementary Fig. 4b). Furthermore, depletion of POLD1 suppressed the ability of Ishikawa, AN3CA and HEC-1A cells to form colonies (Supplementary Fig. 4c). EdU incorporation assays revealed that the fraction of DNA-replicating cells was reduced by silencing POLD1 expression in Ishikawa, AN3CA and HEC-1A cells (Fig. 6c and Supplementary Fig. 4d). Flow cytometry analysis demonstrated that SNRPB knockdown resulted in cell cycle arrest at the G1 phase and induced the apoptosis of Ishikawa cells (Fig. 6d–g). Endometrial cancer cells were tested for their metastatic capacity via Transwell assays in which POLD1 was silenced, and the results demonstrated that POLD1 depletion inhibited the migratory and invasive capacities of Ishikawa, AN3CA and HEC-1A cells (Supplementary Fig. 4e, f). To demonstrate the oncogenic biological functions of the splicing factor SNRPB through the regulation of POLD1 expression, POLD1 siRNA was transfected into SNRPB-overexpressing Ishikawa and AN3CA cells. POLD1 knockdown impaired the increase in growth rate and colony formation capacity induced by SNRPB in endometrial cells (Fig. 6h, i). Hence, POLD1 might play a crucial role as the downstream target responsible for facilitating the oncogenic functions of SNRPB in endometrial cancer cells.

### SNRPB regulates intron 22 retention in POLD1 pre-mRNA in endometrial cancer cells

**Fig. 7:**
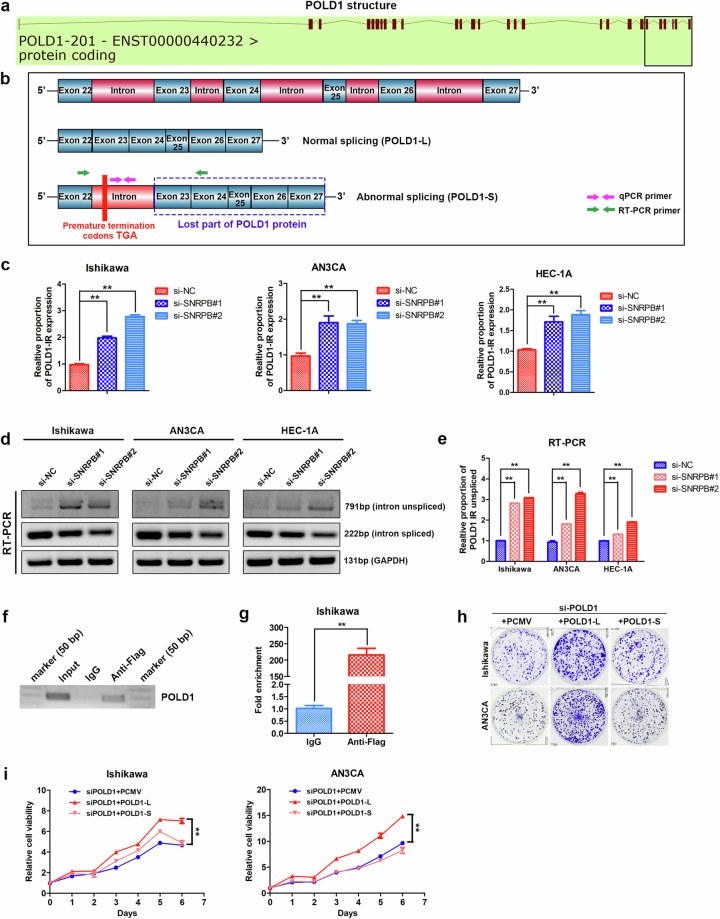
SNRPB regulates intron 22 retention in POLD1 pre-mRNA in endometrial cancer cells. **a** Details of the POLD1 transcript were obtained from the Ensembl database. The black box indicates the position of retained intron 22 in POLD1 after SNRPB was knocked down in Ishikawa cells. **b** A zoomed view indicating the position of the retained introns in POLD1 and the products of the normal and abnormal splicing of POLD1. **c** qPCR was used to detect the relative expression of unspliced intron 22 of the POLD1 transcript (POLD1-S) after SNRPB depletion in endometrial cancer cells via specific primers against intron 22. **d** Specific primers were designed within exon 22 and exon 24 spanning intron 22, and RT‒PCR was performed to distinguish the presence or absence of intron 22 of POLD1. GAPDH served as the endogenous control. **e** The proportion of intron 22 retained in POLD1 after SNRPB silencing in endometrial cancer cells according to the data from (**d**). **f** RNA immunoprecipitation was performed to capture RNA from extracts from Ishikawa cells expressing SNRPB-Flag via an anti-Flag antibody (IgG as a control). RT‒PCR was applied to determine the relative enrichment of POLD1 mRNA levels following the RIP assay. **g** qPCR was applied to determine the relative enrichment of POLD1 mRNA levels following the RIP assay. **h** The clonogenic assay was performed to assess the effects of POLD1-L and POLD1-S overexpression on the colony formation abilities of Ishikawa and AN3CA cells with POLD1 silencing. **i** MTT cell proliferation curves were performed to assess the effects of POLD1-L and POLD1-S overexpression on the proliferation abilities of Ishikawa and AN3CA cells with POLD1 silencing. The *P* value was obtained via an unpaired *t*-test. *P < 0.05, **P < 0.01.

To understand the mechanism responsible for SNRPB-mediated regulation of POLD1 intron retention, we obtained details about the POLD1 transcript from the Ensembl database. The black box in Fig. 7a indicates the position of the retained intron 22 in POLD1 after the knockdown of SNRPB in Ishikawa cells. A zoomed-in view indicates the position of the retained introns in POLD1, and the products of the normal and abnormal splicing of POLD1 are shown schematically in Fig. 7b. A PCNA interaction motif between amino acids 1001 and 1005 in the C-terminal domain of POLD1 was identified, and this motif is essential for its enzyme activity^30^. The product of intron 22 retention in POLD1 harbored premature termination codons, which led to the loss of the PCNA binding site.

Primers were designed for intron 22 to examine the expression of unspliced intron 22 of the POLD1 transcript (abnormal splicing transcript POLD1-S) after SNRPB depletion in endometrial cancer cells. The relative proportion of POLD1-S transcript expression containing intron 22 was elevated after SNRPB inhibition in Ishikawa, AN3CA and HEC-1A cells (Fig. 7c). Specific primers were designed within exon 22 and exon 24 spanning intron 22, and RT‒PCR was performed to distinguish the presence or absence of intron 22. The expression of the POLD1-S transcript with intron 22 retention increased, and the expression of POLD1-L without intron 22 retention (normal splicing transcript) decreased after SNRPB silencing in Ishikawa, AN3CA and HEC-1A cells (Fig. 7d). SNRPB depletion increased the proportion of intron 22 retention in POLD1 in endometrial cancer cells (Fig. 7e). Furthermore, a RIP experiment was performed to obtain RNA from extracts from Ishikawa cells expressing SNRPB-Flag via an anti-Flag antibody (IgG as a control). The relative enrichment in POLD1 mRNA levels was evaluated by RT‒PCR and qPCR following the RIP assay. These results demonstrated that POLD1 mRNA was significantly enriched by the anti-Flag antibody compared with the negative control IgG (Fig. 7f, g).

To study the effects of different POLD1 transcripts (POLD1-L and POLD1-S) on the biological behavior of endometrial cancer cells, overexpression vectors for the full-length POLD1-L transcript (NM_002691) and truncated POLD1-S transcript (amino acids 1 and 940, from the start codon to exon 22) were constructed. MTT cell proliferation curves and clonogenic assays of POLD1-L- and POLD1-S-overexpressing Ishikawa and AN3CA cells with POLD1 silencing were performed, and the findings revealed that POLD1-L promoted the growth and colony formation of endometrial cancer cells (Fig. 7h, i). However, compared with POLD1-L overexpression, POLD1-S overexpression reduced proliferation and colony formation (Fig. 7h, i). These results are consistent with the effects of POLD1 on proliferative and metastatic capacities and are dependent mainly on the POLD1 C-terminus^25^. These data indicated that the splicing factor SNRPB promoted the effective splicing of POLD1 pre-mRNA in endometrial cancer cells.

### ASO-mediated SNRPB silencing inhibits the proliferative capacity of endometrial cancer cells

**Fig. 8:**
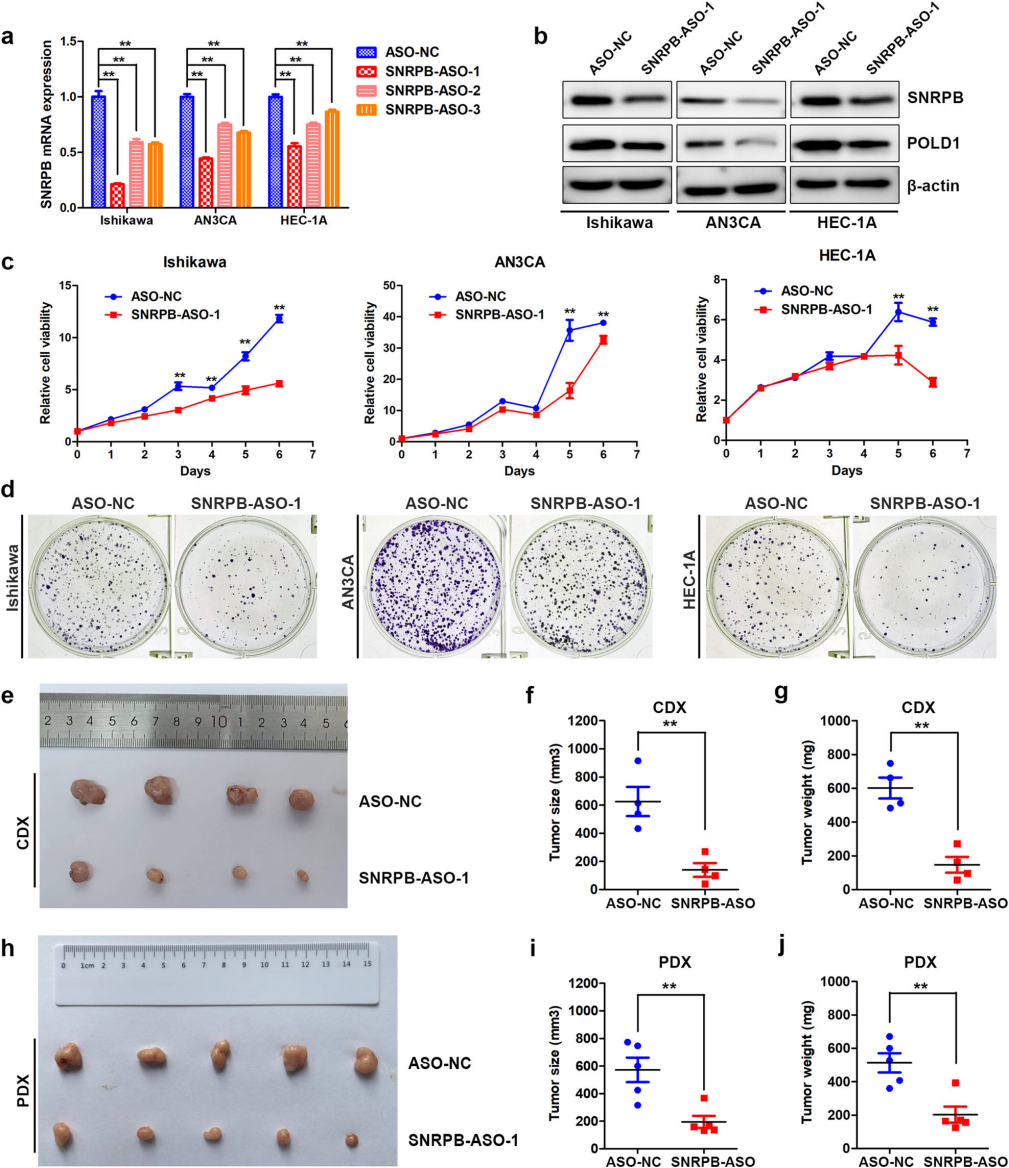
ASO-mediated SNRPB inhibition decreases the proliferation ability of CDX and PDX models of endometrial cancer. **a** A qPCR analysis of the effect of SNRPB-specific ASOs on SNRPB mRNA expression in Ishikawa, AN3CA and HEC-1A cells (*n* = 3 biologically independent samples). **b** Western blot analysis of the effect of SNRPB-ASO on SNRPB and POLD1 protein levels in endometrial cancer cells. **c**,**d** MTT cell proliferation curves (**c**) and clonogenic assays (**d**) were performed to assess the effects of SNRPB-ASO on the proliferation and colony formation abilities of endometrial cancer cells. **e** Photographs of endometrial cancer CDX tumors removed from sacrificed nude mice in the SNRPB-ASO group and ASO-NC group (*n* = 4 mice per group). **f**, **g** The volume (**f**) and weight (**g**) of the endometrial cancer CDX tumors were measured and calculated in the SNRPB-ASO group and ASO-NC group. **h** Photographs of endometrial cancer PDX tumors removed from killed NCG mice in the SNRPB-ASO group and ASO-NC group (*n* = 5 mice per group). **i**, **j** The volume (**i**) and weight (**j**) of the endometrial cancer PDX tumors were measured and calculated in the SNRPB-ASO group and ASO-NC group. The *P* value was obtained via an unpaired *t*-test. *P < 0.05, **P < 0.01.

ASOs have emerged as promising therapeutic approaches in personalized medicine. In this study, ASOs targeting SNRPB were designed and synthesized by GenePharma. Endometrial cancer cells were transfected with these ASOs via Lipofectamine 2000. qPCR was employed to assess the impact of SNRPB-ASOs on its mRNA expression, revealing a significant decrease in SNRPB mRNA levels with SNRPB-ASO-1 in all three endometrial cancer cell lines (Fig. 8a). Furthermore, western blot analysis confirmed that SNRPB-ASO-1 also reduced the protein expression of SNRPB (Fig. 8b and Supplementary Fig. 5a). MTT proliferation and colony formation assays were used to assess the impact of SNRPB-ASO-1 on the malignant behavior of endometrial cancer. These findings indicated that SNRPB-ASO-1 effectively suppressed the proliferation and colony formation abilities of endometrial cancer cells (Fig. 8c, d). However, the SNRPB-specific ASO did not significantly affect human endometrial stromal cell growth or metastasis (Supplementary Fig. 5b–d). To further investigate the antitumor efficacy of SNRPB-ASO in vivo against endometrial cancer, we established a CDX model by subcutaneously injecting Ishikawa cells into nude mice. Following tumor establishment, we administered intratumoral injections of SNRPB-ASO or a negative control ASO-NC to evaluate their therapeutic effects, with four mice allocated to each group (Fig. 8e). The findings revealed that both tumor volume and weight were significantly lower in the SNRPB-ASO treatment group than in the ASO-NC group (Fig. 8f, g). Next, a PDX model was established, and intratumoral administration of SNRPB-ASOs (*n* = 5) was compared with that of ASO-NCs (*n* = 5) to evaluate therapeutic efficacy in the PDX model (Fig. 8h). The results revealed significant reductions in tumor weight and volume in the SNRPB-ASO treatment group (Fig. 8i, j). Taken together, these results suggested that ASO-mediated SNRPB inhibition decreased the proliferative capacity of endometrial cancer cells both in vitro and in vivo.

## Discussion

The dysregulation of RNA splicing is a molecular characteristic of almost all types of tumors^31^. Splicing factors play essential roles in tumorigenesis and the development of human malignancies^32^. SNRPB is overexpressed in multiple human cancers and modulates multiple processes, such as cell proliferation, migration, invasion, cisplatin resistance and stemness^14,17,20^. In this study, we find that SNRPB was highly expressed in frozen or paraffin-embedded endometrial cancer samples and that high SNRPB expression was associated with unfavorable clinical outcomes in patients with endometrial cancer. In addition, SNRPB silencing suppressed the growth rate and metastasis of endometrial cancer cells. Our research revealed that the splicing factor SNRPB functions as an oncogenic driver in endometrial cancer. Hence, targeting the splicing factor SNRPB represents a new strategy for the treatment of endometrial cancer.

Alternative splicing plays an important role in the initiation and progression of endometrial cancer. The splicing factor SF3B1 is highly expressed in endometrial tissues, and SF3B1 silencing impaired the proliferation, migration and invasion abilities of tumor cells. Furthermore, the suppression of SF3B1 has been shown to induce G2/M arrest in endometrial cancer cells and impede the maturation of KSR2 pre-mRNA^5^. In this study, we find that SNRPB was overexpressed in endometrial cancer tissues and that high SNRPB expression indicated an unfavorable prognosis in patients with endometrial cancer. SNRPB promoted the malignant progression of endometrial cancer cells through regulating POLD1 expression. Low expression of miR-654-5p contributed to high SNRPB expression through direct binding to its 3′-UTR, and these results are consistent with our previous findings in ovarian cancer^14^. The relationship between miR-654-5p and SNRPB expression extends beyond endometrial cancer, suggesting the broader applicability of this regulatory interaction between miR-654-5p and SNRPB.

ASOs represent a novel class of highly specific nucleic acids engineered to silence target genes^33^. ASOs targeting SNORD14E have been shown to attenuate tumor cell proliferation and metastasis and induce apoptosis while impeding tumor growth in an endometrial cancer xenograft mouse model^34^. In this study, ASOs targeting SNRPB were developed and validated, with subsequent inhibition of SNRPB resulting in reduced proliferation in CDX and PDX mouse models. These findings suggest that ASO-mediated suppression of SNRPB may be a promising therapeutic approach for the treatment of endometrial cancer.

The POLD1 gene is located on chromosome 19q13.33, and its primary transcript (NM_002691.3) consists of 27 exons and produces a protein called p125 (1107 amino acids), which serves as the catalytic subunit of DNA polymerase delta (Polδ)^35^. POLD1 plays a central role in DNA replication and repair in cancers^29^. POLD1 stabilizes MYC and leads to faster cell growth and metastasis in bladder cancer cells^25^. POLD1 knockdown impedes cell proliferation and DNA synthesis and leads to cell cycle arrest^36^. POLD1 is a biomarker of poor clinical outcomes and is associated with proliferation ability and the immune response in clear cell renal cell carcinomas^37^. In this study, we find that silencing the expression of the splicing factor SNRPB decreased POLD1 expression levels in endometrial cancer cells, and POLD1 was identified as a critical downstream target of SNRPB in endometrial cancer cells.

Most genes generate proteomic complexity through alternative splicing, and intron retention is a unique mechanism for reducing gene expression^38^. Premature termination codons typically occur in intron-retaining transcripts, and aberrant transcripts are often removed by surveillance mechanisms^38^. Intron-retaining transcripts can also be translated to produce truncated proteins due to in-frame stop codons^39^. There are two major domains in p125: a catalytic core domain located at the N-terminus containing polymerases and exonucleases and a metal-binding domain situated at the C-terminus^40^. The PCNA interaction motif is present between amino acids 1001 and 1005 (exon 24) in the C-terminal domain of POLD1, and this motif is essential for its enzyme activity^30,40^. In this study, we reveal that POLD1 intron 22 retention was induced after SNRPB depletion in endometrial cancer cells. The retained intron 22 of POLD1 created a premature termination codon, leading to the absence of amino acids (941 and 1107) from exon 23 to exon 27 and the loss of the site of interaction with PCNA. Multiple PCNA-interacting protein domains are present in the Polδ complex, but POLD1 is the key partner. The interaction between POLD1 and PCNA is necessary for the activity and processivity of POLD1^40^. Here, we propose a potential new strategy for controlling the interaction between POLD1 and PCNA through the splicing factor SNRPB in endometrial cancer cells.

Through our research, the splicing factor SNRPB was found to be overexpressed in patients with endometrial cancer and associated with an unfavorable clinical outcome. Loss of SNRPB reduced endometrial cancer cell growth and invasion by regulating the retention of POLD1 introns. Intron retention decreases the expression and normal function of the canonical POLD1 gene post-transcriptionally in endometrial cancer cells. Low expression of miR-654-5p promoted the overexpression of SNRPB through direct binding to its 3′-UTR in endometrial cancer cells. Thus, the oncogenic driver SNRPB–POLD1 axis represents an interesting therapeutic target for endometrial cancer treatment. ASO-mediated silencing of SNRPB might provide an effective treatment strategy for treating patients with endometrial cancer.

## Supplementary Information

Supplementary Information

Supplementary Table 3

Original images of western blotting.

## Data Availability

The datasets used and/or analyzed during the current study are available from the corresponding author upon reasonable request.
