# Supplementary material for: Targeting the splicing factor SNRPB inhibits endometrial cancer progression by retaining the POLD1 intron
Source: Exp Mol Med. 2025 Feb 5;57(2):420–35. doi: 10.1038/s12276-025-01407-2 (PMC11873159; doi:10.1038/s12276-025-01407-2)
Supplement: Supplementary file 1 — Supplementary Information [file 12276_2025_1407_MOESM1_ESM.pdf]

Supplementary Figures

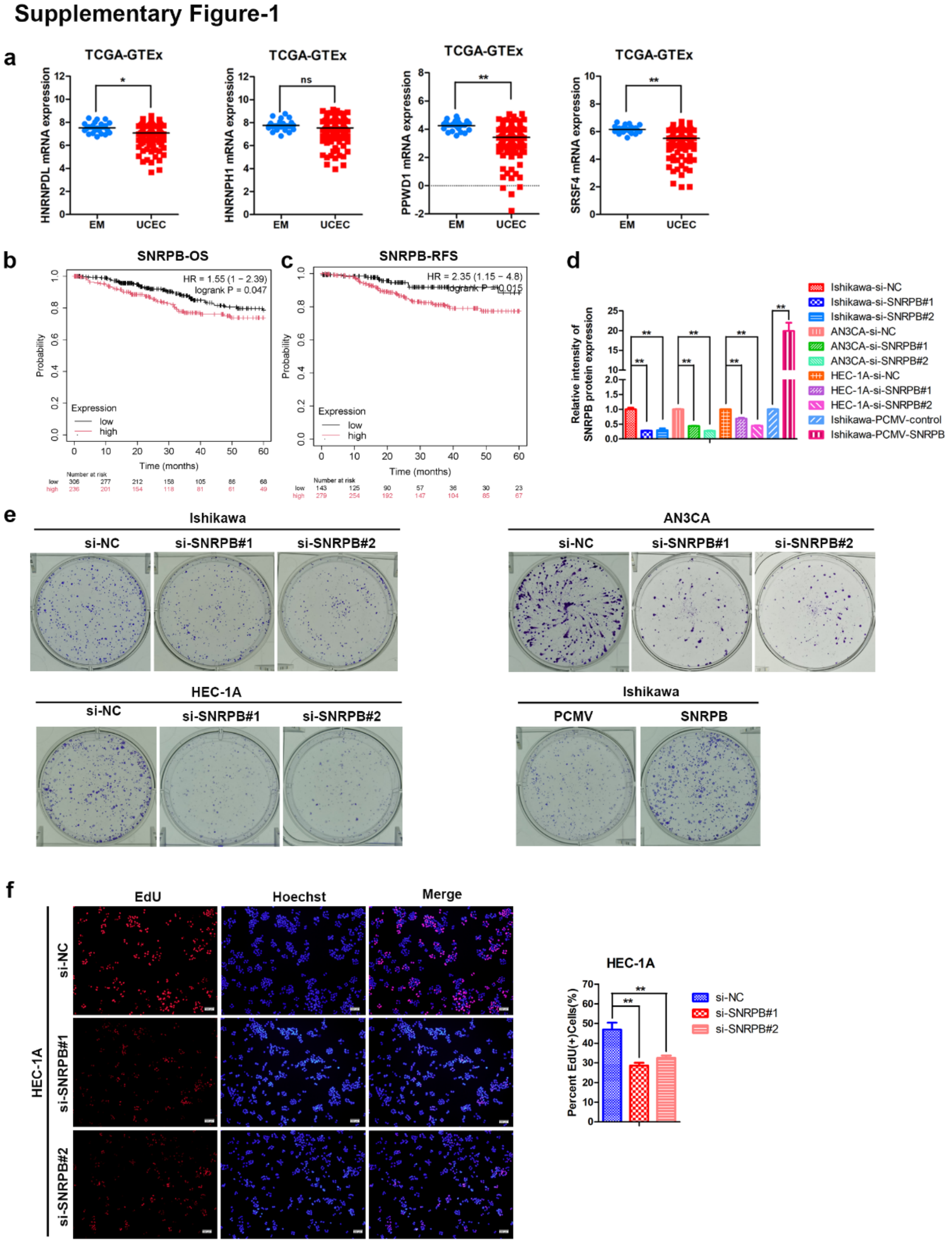

Supplementary Fig. 1. (a) Differential expression analysis of HNRNPDL, HNRNPH1, PPWD1, and SRSF4 between endometrial cancer (n=181) and normal endometrium (n=23) tissues using the data from TCGA-GTEx. (b-c) The effect of SNRPB on the overall survival (low expression, n=306; high expression, n=236) and relapse-free

survival (low expression, n=143; high expression, n=279) of endometrial cancer patients was analyzed using the Kaplan–Meier plotter online tool. (d) Quantification of western blotting bands derived from Fig.2b. (e) The colony formation assay was used to evaluate the impact of SNRPB knockdown in Ishikawa, AN3CA, and HEC-1A cells and overexpression in Ishikawa cells on the viability of endometrial cancer cells (n=3 biologically independent samples). (f) The fraction of DNA-replicating cells was reduced by silencing SNRPB in HEC-1A cells were subjected to EdU incorporation assay (n=3 biologically independent samples). P values were obtained by unpaired t tests (a, d and f) or log-rank tests (b and c).

Supplementary Figure-2

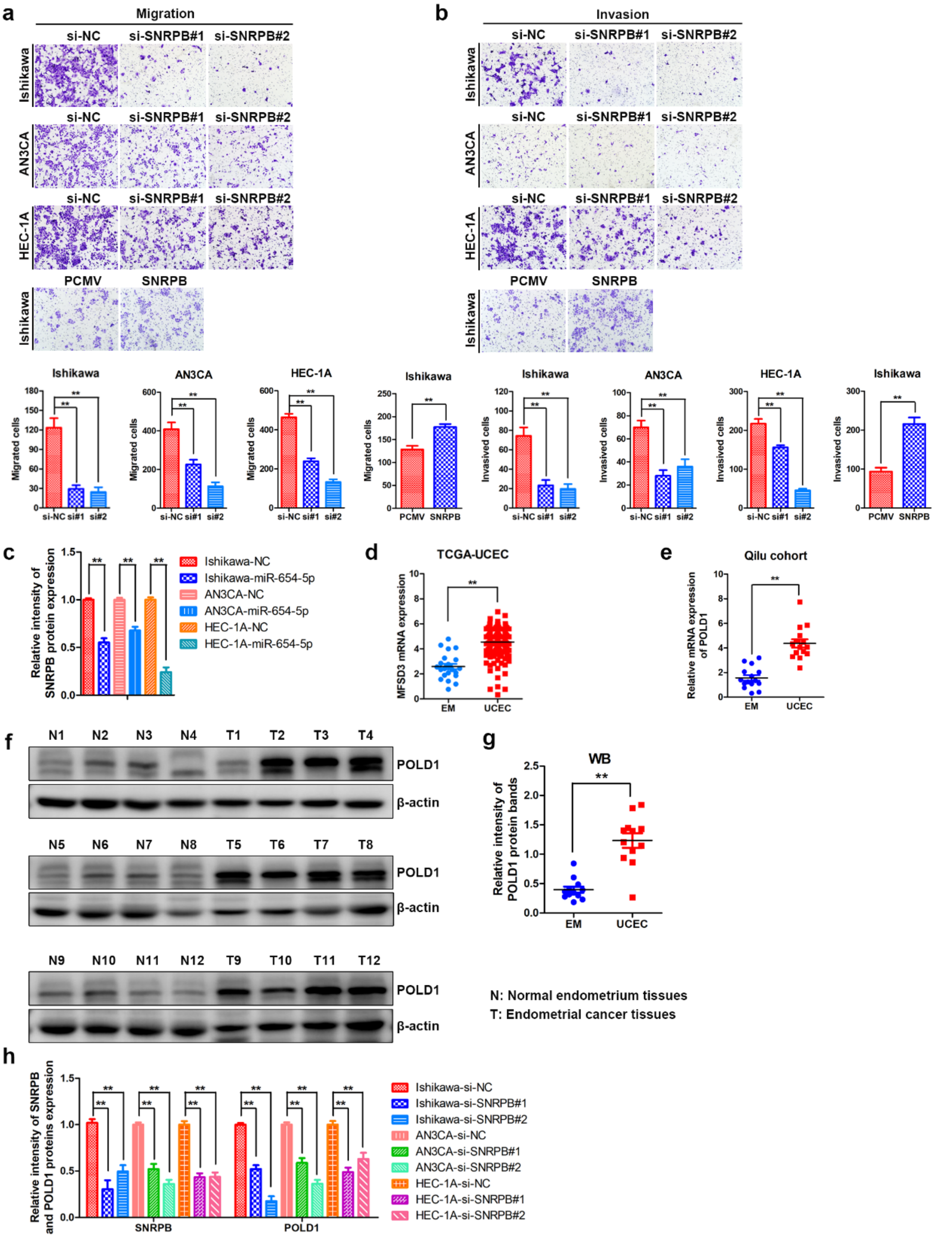

Supplementary Fig. 2. (a-b) Transwell migration and Matrigel-coated Transwell invasion assays were conducted to examine the impact of SNRPB silencing on the cell migration and invasion capabilities of Ishikawa, AN3CA, and HEC-1A cells (n=3 biologically independent samples). (c) Quantification of western blotting bands derived

from Fig. 4d. (d) Differential expression analysis of MFSD3 between endometrial cancer (n=181) and normal endometrium (n=23) tissues using the data from TCGA-UCEC. (e) qPCR analysis of POLD1 mRNA expression in fresh-frozen tissues from endometrial cancer patients (n=15) and normal endometrium (n=15). (f) Western blotting analysis of POLD1 protein expression in fresh-frozen tissues from endometrial cancer patients (T, n=12) and normal endometrium (N, n=12). (g) Quantification of western blotting bands derived from Supplementary Fig. 2f. (h) Quantification of western blotting bands derived from Fig. 5l. The P value was obtained by an unpaired t test. *P < 0.05, **P < 0.01.

Supplementary Figure-3

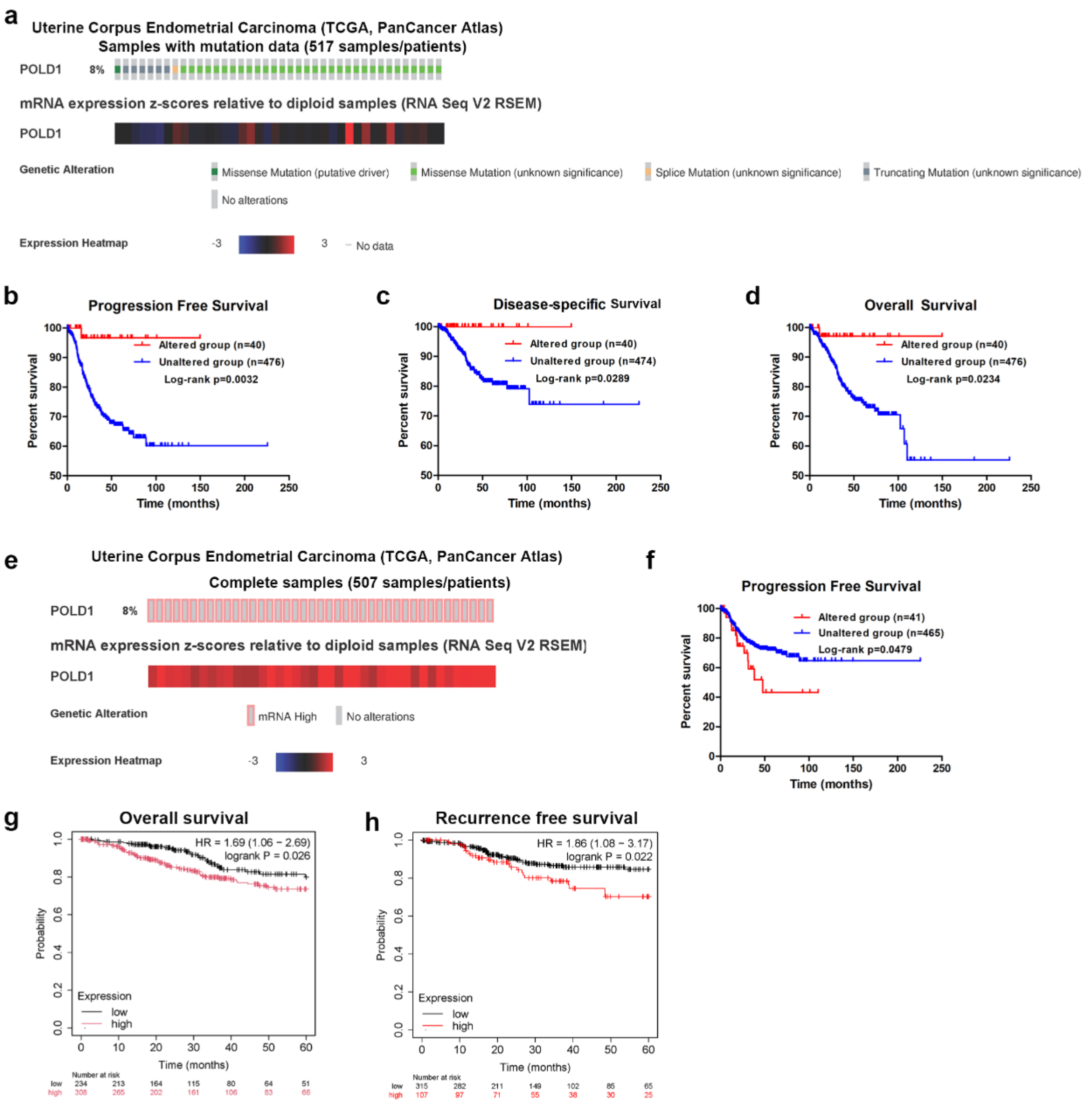

Supplementary Fig. 3. (a) The POLD1 mutation ratio was evaluated in uterine corpus endometrial carcinoma cases (n=517) from the TCGA, Nature 2013 and the TCGA PanCancer atlas. (b) The effect of POLD1 mutation on the Progression Free Survival (PFS) was determined in endometrial cancer cases (Altered group, n=40; Unaltered group, n=476) from the TCGA PanCancer atlas. (c) The effect of POLD1 mutation on the Disease Specific Survival (DSS) was determined in endometrial cancer cases (Altered group, n=40; Unaltered group, n=474) from the TCGA PanCancer atlas. (d) The effect of POLD1 mutation on the Overall Survival (OS) was determined in

endometrial cancer cases (Altered group, n=40; Unaltered group, n=476) from the TCGA PanCancer atlas. (e) The mRNA expression of POLD1 in endometrial cancer patients (n=507) was determined using the TCGA PanCancer atlas database. (f) The effect of POLD1 mRNA expression on PFS in endometrial cancer patient (Altered group, n=41; Unaltered group, n=465) data from the TCGA PanCancer atlas. (g) The relationship between POLD1 mRNA expression and OS in endometrial cancer patients (low expression, n=234; high expression, n=308) was examined using the KMplot website. (h) The relationship between POLD1 mRNA expression and RFS in endometrial cancer patients (low expression, n=315; high expression, n=107) was examined using the KMplot website. The P value was obtained by log-rank tests. *$P < 0.05$, **$P < 0.01$.

Supplementary Figure-4

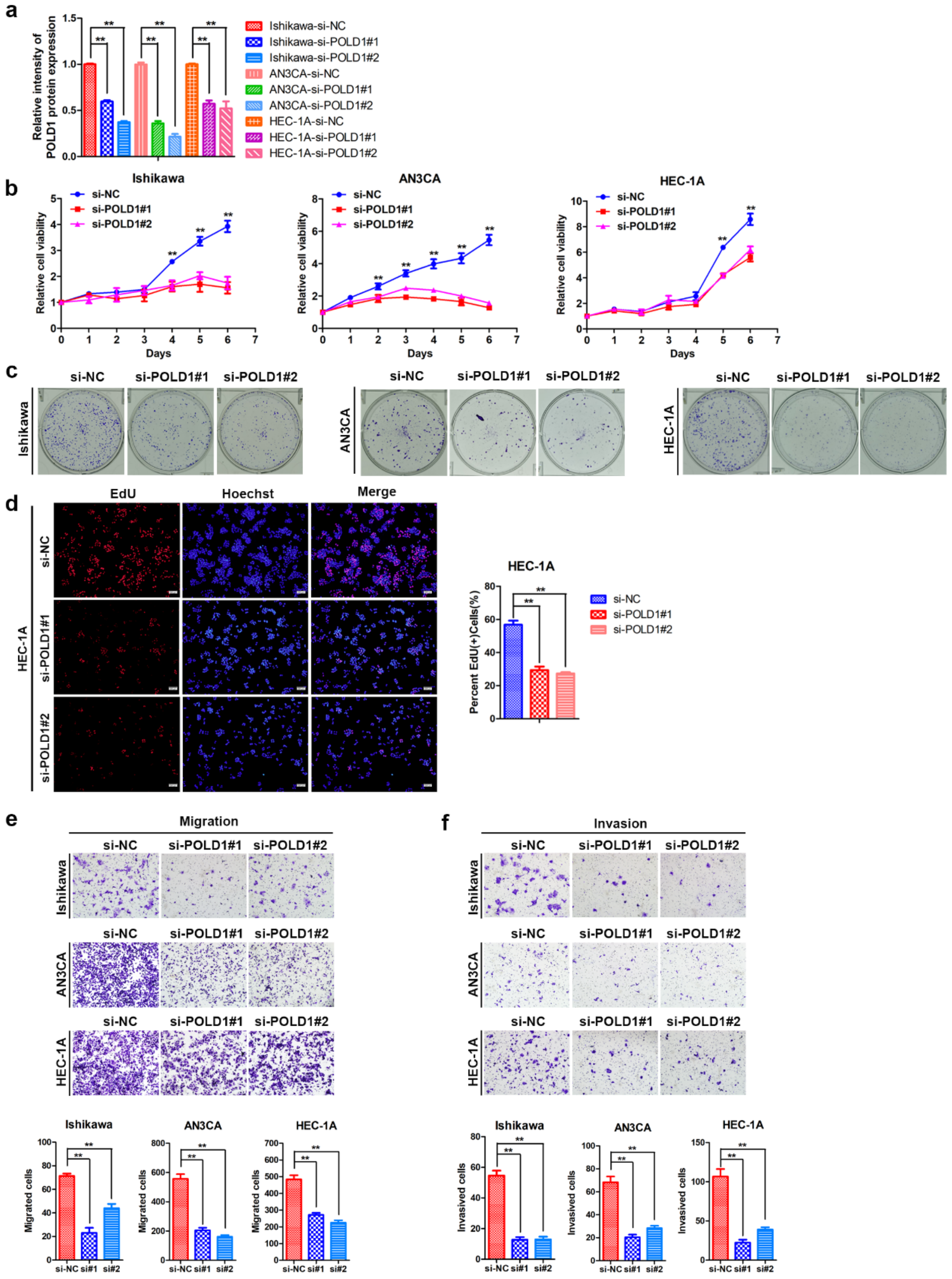

Supplementary Fig. 4. (a) Quantification of western blotting bands derived from Fig. 6b. (b) The effect of POLD1 knockdown on proliferation ability was evaluated by the growth curve assay in Ishikawa, AN3CA and HEC-1A cells. (c) A colony formation

assay was used to evaluate the impact of POLD1 knockdown on the viability of endometrial cancer cells (n=3 biologically independent samples). (d) The fraction of DNA-replicating cells was reduced by silencing POLD1 in HEC-1A cells were subjected to EdU incorporation assay (n=3 biologically independent samples). (e-f) Transwell assays were performed to determine the impact of POLD1 silencing on the migration and invasion of Ishikawa, AN3CA, and HEC-1A cells. The P value was obtained by an unpaired t test. *P < 0.05, **P < 0.01.

Supplementary Figure-5

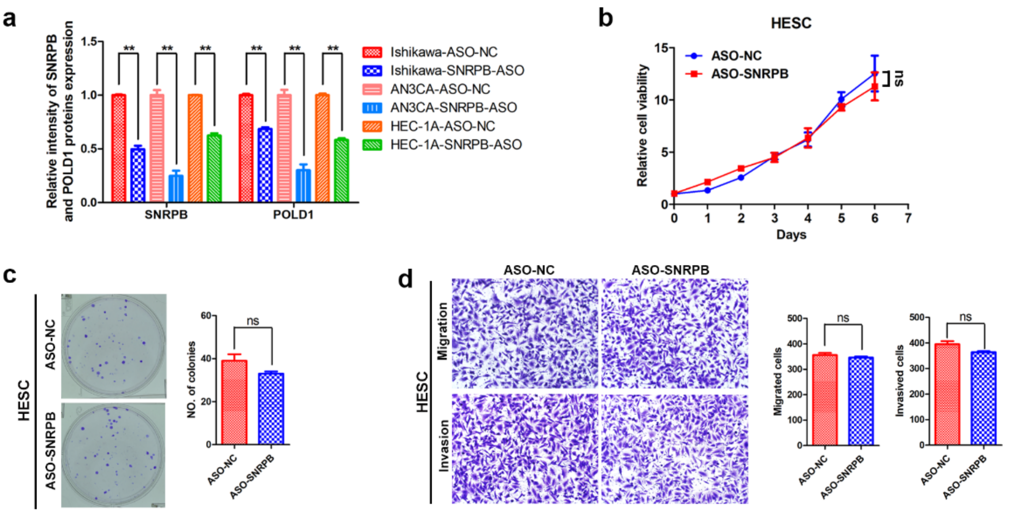

Supplementary Fig. 5. (a) Quantification of western blotting bands derived from Fig. 8b. (b-c) MTT cell proliferation curves and clonogenic assays were performed to assess the effect of SNRPB-ASO on the proliferation and colony formation abilities in Human Endometrial Stromal Cells (HESC) cells. (d) Transwell assays were performed to determine the impact of SNRPB-ASO on the migration and invasion of HESC cells. The P value was obtained by an unpaired t test. *P < 0.05, **P < 0.01.

Supplementary Table-1. siRNA sequences used in this study.

siRNA	sequence
si-SNRPB#1	5′-CAAGCCAAAGAACUCCAAATT-3′
si-SNRPB#2	5′-UCCCAAAGAUACUGGUAUUTT-3′
si-POLD1#1	5′-GUUGGAGAUUGACCAUUAUTT-3′
si-POLD1#2	5′-CCGGUUACAACAUCCAGAATT-3′

Supplementary Table-2. Antisense oligonucleotides sequences used in this study.

ASO	sequence
SNRPB-ASO-1	TAAACCAGTTTCATAGGCC
SNRPB-ASO-2	TAAGAAACAAACAGGTCTG
SNRPB-ASO-3	ATACCAGTATCTTTGGGAG

Supplementary Table-3. Clinical information of endometrial cancer patients.
