# Supplementary figures and images for: Targeting the splicing factor SNRPB inhibits endometrial cancer progression by retaining the POLD1 intron
Source: Exp Mol Med. 2025 Feb 5;57(2):420–35. doi: 10.1038/s12276-025-01407-2 (PMC11873159; doi:10.1038/s12276-025-01407-2)

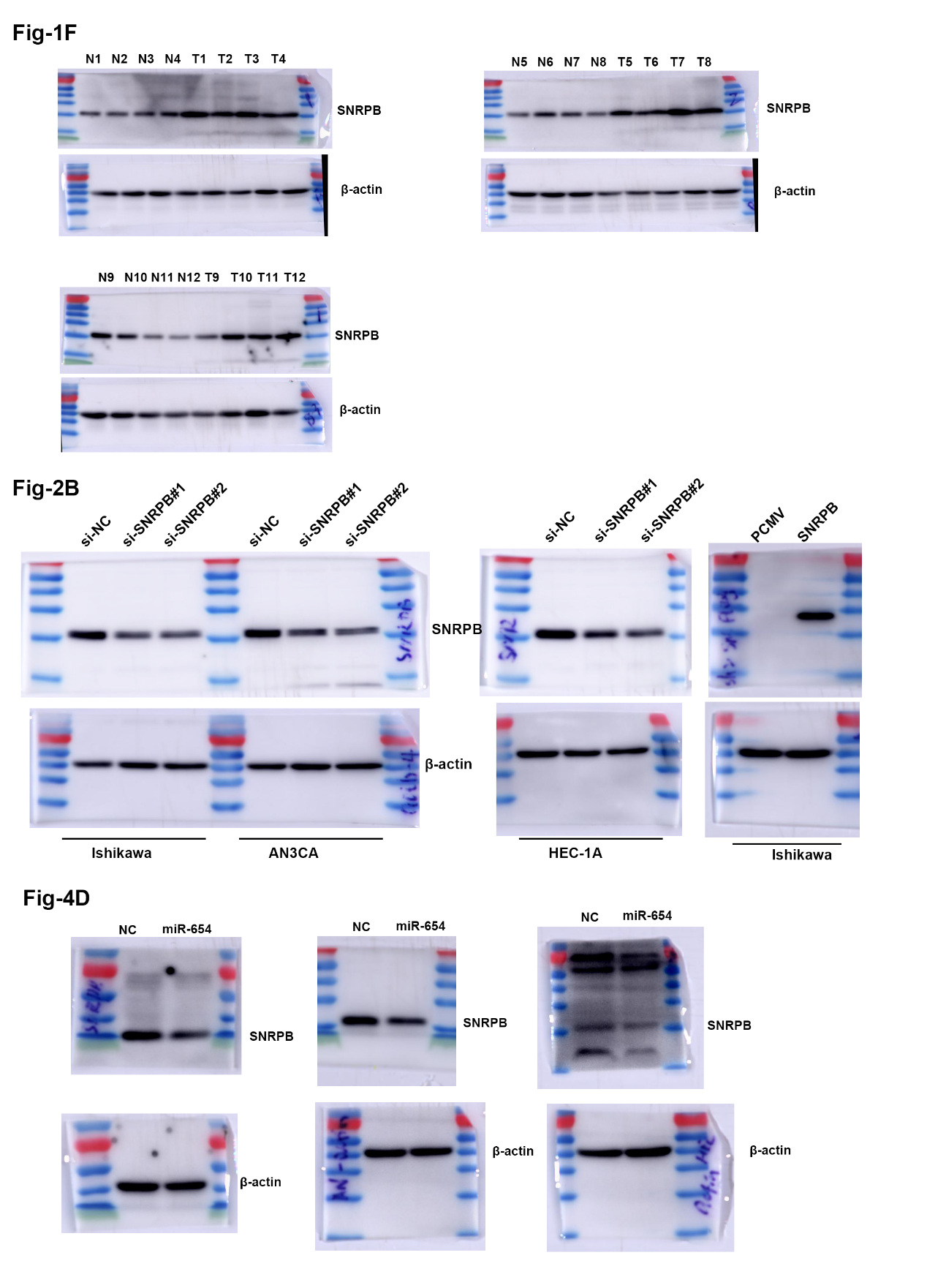


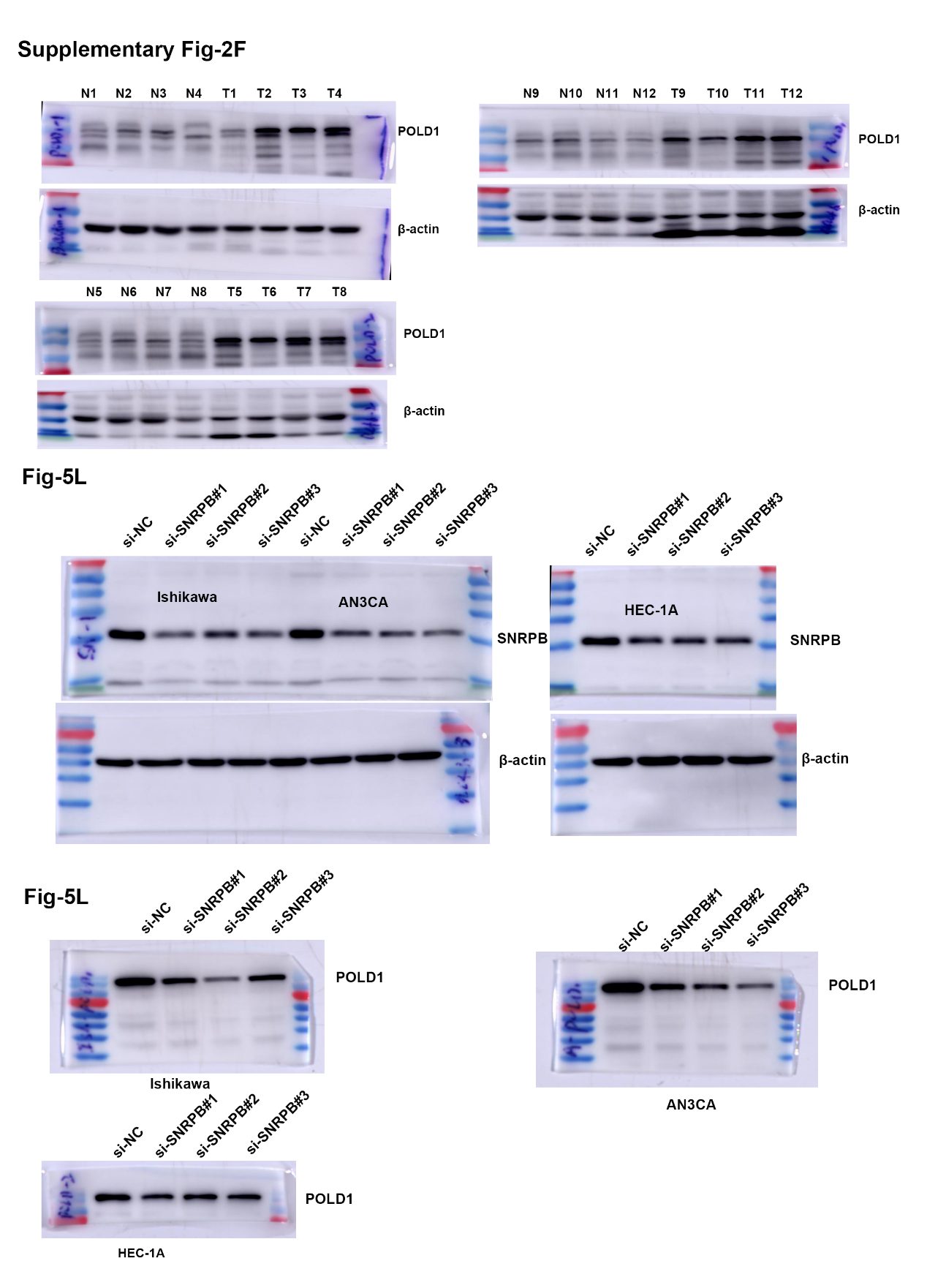


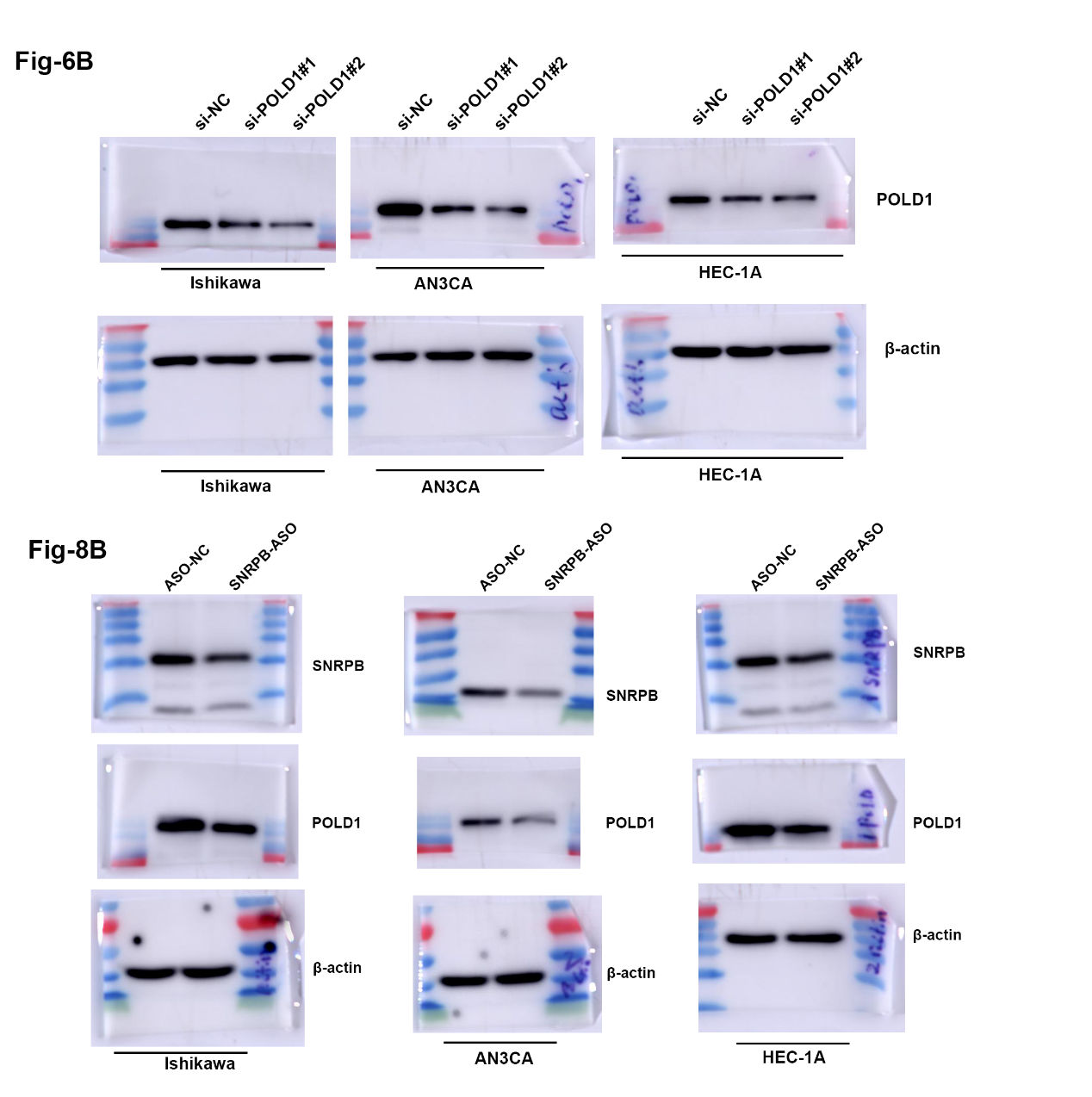

Supplement: Supplementary file 3 — Original images of western blotting. [file 12276_2025_1407_MOESM3_ESM.docx]
